# How Effective is Integrated Vector Management Against Malaria and Lymphatic Filariasis Where the Diseases Are Transmitted by the Same Vector?

**DOI:** 10.1371/journal.pntd.0003393

**Published:** 2014-12-11

**Authors:** Christopher M. Stone, Steve W. Lindsay, Nakul Chitnis

**Affiliations:** 1 Department of Epidemiology and Public Health, Swiss Tropical and Public Health Institute, Basel, Switzerland; 2 University of Basel, Basel, Switzerland; 3 School of Biological and Biomedical Sciences, Durham University, Durham, United Kingdom; University of Oklahoma Health Sciences Center, United States of America

## Abstract

**Background:**

The opportunity to integrate vector management across multiple vector-borne diseases is particularly plausible for malaria and lymphatic filariasis (LF) control where both diseases are transmitted by the same vector. To date most examples of integrated control targeting these diseases have been unanticipated consequences of malaria vector control, rather than planned strategies that aim to maximize the efficacy and take the complex ecological and biological interactions between the two diseases into account.

**Methodology/Principal Findings:**

We developed a general model of malaria and LF transmission and derived expressions for the basic reproductive number (R_0_) for each disease. Transmission of both diseases was most sensitive to vector mortality and biting rate. Simulating different levels of coverage of long lasting-insecticidal nets (LLINs) and larval control confirms the effectiveness of these interventions for the control of both diseases. When LF was maintained near the critical density of mosquitoes, minor levels of vector control (8% coverage of LLINs or treatment of 20% of larval sites) were sufficient to eliminate the disease. Malaria had a far greater R_0_ and required a 90% population coverage of LLINs in order to eliminate it. When the mosquito density was doubled, 36% and 58% coverage of LLINs and larval control, respectively, were required for LF elimination; and malaria elimination was possible with a combined coverage of 78% of LLINs and larval control.

**Conclusions/Significance:**

Despite the low level of vector control required to eliminate LF, simulations suggest that prevalence of LF will decrease at a slower rate than malaria, even at high levels of coverage. If representative of field situations, integrated management should take into account not only how malaria control can facilitate filariasis elimination, but strike a balance between the high levels of coverage of (multiple) interventions required for malaria with the long duration predicted to be required for filariasis elimination.

## Author Summary

Integrated vector management aims to optimize efficacy and make better use of available funds, including targeting multiple diseases, using one or more interventions. However, we have relatively poor understanding of the programmatic demands that arise when controlling two diseases. For instance, does the intensity, duration of deployment, or type of intervention most suitable for each disease overlap or clash? We developed a mathematical model to explore these issues for the example of the vector-borne diseases malaria and lymphatic filariasis. Since the causative agents of these diseases are transmitted by the same mosquito species in certain areas, there is clear potential for an integrated approach using long-lasting insecticidal nets (LLINs) or larval source management. We found that the transmission potential of both malaria and LF is most sensitive to changes in mosquito survivorship and the duration of the feeding cycle, supporting the usefulness of LLINs. In areas where both diseases occur, malaria elimination was predicted to require high levels of both LLINs and larval source management, whereas either intervention at a low intensity was sufficient to eliminate LF, if maintained for a longer period. This highlights that integrated control programs should be flexible and dynamic in order to accommodate these demands.

## Introduction

Vector control continues to play a major role in ameliorating the burden of vector-borne diseases, such as malaria, for instance through the use of long lasting insecticidal nets (LLINs) [1]. As progress is made toward meeting the goals of local elimination and global eradication that have been set for a number of vector-borne diseases, there is a need in many parts of the world to further reduce the intensity of transmission and make better use of existing funds over the long term [2], [3]. Integrated vector management (IVM) is seen as a way to make rational decisions about the choice of vector control tools, improve (cost-)effectiveness and sustainability of control and limit the use of insecticides based on an understanding of local ecological conditions [2], [4]. Within IVM there are two broad approaches: one which uses a combination of interventions against a single disease and one which uses one or more interventions against more than one vector-borne disease. Since there are few field studies that report the impact of vector control interventions on more than one vector-borne disease, we use mathematical modelling to explore how common vector control tools could impact two diseases transmitted by one vector species.

Malaria and lymphatic filariasis (LF), both transmitted by the same vectors in rural Africa, serve as an examplar of the potential benefits and complications of IVM. The potential for integrating control across both diseases stems from their broad geographic overlap, shared vectors across much of this range, and susceptibility to the same interventions [2], [5]. This is particularly relevant for the poorest countries where the burden due to these diseases remains the highest. In Africa, particularly in areas where *Loa loa* co-occurs, mass drug administration programmes to clear *Wuchereria bancrofti* microfilariae from the human population face the challenge that the most effective anti-helminthics are contraindicated and vector control may have to be relied on more heavily [6]. In areas with the highest malaria burden due to *Plasmodium falciparum* infections, and especially in rural areas where malaria is spread by the efficient and anthropophilic vectors *Anopheles gambiae s.l.* and *An. funestus*, that are also primary vectors of *W. bancrofti* [7], current control measures may not be sufficient to interrupt transmission, and increasing worries about insecticide resistance highlight the need for an efficient, sustainable, and well thought out approach to controlling multiple diseases [8].

To date, synergy between malaria and LF control programmes has been mostly in the form of almost accidental side-effects of malaria control on filariasis transmission. A notable example being the Solomon Islands malaria eradication initiative commenced in 1960, where both parasites were transmitted by *An. farauti* and *An. koliensis* [9], [10], [11]. Whilst indoor residual spraying for 10 years failed to eradicate malaria, it resulted in the disappearance of LF from the island. Bed nets have likewise been shown to affect transmission of LF. Use of untreated bed nets in one village in Papua New Guinea was associated with a reduction in the proportion of vectors harbouring infective larvae from 5.38% to 1.62% [12]. Despite the large number of studies that have investigated the impact of LLINs on malaria there have been few that investigated their effect on LF. In Kenya the introduction of treated nets lowered the number of indoor-resting *An. gambiae s.l.* and *An. funestus* and reduced the human blood index of *Culex quinquefasciatius* [13]. Similarly the proportion of infected *An. punctulatus* decreased from 1.8% to 0.4% after the distribution of LLINs in Papua New Guinea, and, importantly, no infective larvae were found [14].

The evidence suggests that interventions directed against malaria vectors may be more effective at controlling LF if their use is sustained at least as long as the average lifespan of adult worms in humans, with estimates ranging from 4–10 years [15], [16]. Central to this is that LF is a far less efficient disease to transmit than malaria [17] requiring a far greater critical density of mosquitoes per human for LF than for malaria. Whilst one study estimated that between 5 and 100 infective bites (depending on the level of immunity of humans) will result in a malaria infection [18], many thousands are required before a patent filarial infection is produced. In Yangon (formerly Rangoon), where *Cx. quinquefasciatus* is the primary vector, it was estimated that an average of 15, 500 infective bites resulted in one microfilaraemic case [19]. The feasibility of interrupting LF transmission where *Anopheles* spp. are the vectors could be enhanced further by the process of facilitation, which is a density-dependent parasite-vector interaction resulting in an increasing yield of infective larvae as the number of microfilariae ingested increases. It has been suggested that this introduces an additional unstable equilibrium point above that associated with worm mating probabilities [20]. Interactions between parasites and mosquitoes in areas co-endemic for malaria and LF, could, potentially, result in perverse effects of control programmes aimed at only one disease [20], [21]. An illustration of this was recently provided, where a higher prevalence of malaria infection in humans was predicted to occur in the absence of LF [22]. The integration of control measures aimed at multiple diseases will thus have to take the different sensitivities of the diseases to interventions, as well as the complexities in transmission dynamics, into account. To explore how multiple interventions could be used as an IVM strategy to control malaria and LF where both parasites are transmitted by the same vector species, we develop a combined mathematical model that takes the interaction between the vector and multiple parasites into account. The model shares characteristics with a recently published model [22], but diverges in a number of areas. The focus initially is on a general anopheline (e.g. *gambiae, funestus,* or *punctulatus* complex) without developing species-specific behavioural characteristics such as the degree of anthropophily or response to interventions. We derive expressions of the basic reproductive numbers, R_0_, of both diseases in the presence and absence of the other disease and calculate the local sensitivity of R_0_ to the transmission parameters it encapsulates in order to gain insight in which parameters contribute most strongly to transmission and therefore make attractive targets for interventions. Additionally, we perform simulations of the impact of LLINs and larval source management to gain insight in the relative efficacy of each for both diseases in the presence and absence of the other. LLINs are used on a massive scale for the control of malaria, particularly in sub-Saharan Africa [23], and are highly effective at controlling vectors entering houses, whilst larval source management can be used as a supplementary intervention in some settings where it will impact both the indoor and outdoor biting population [24]. This study is to our knowledge the first to consider how one or more interventions can impact multiple vector-borne diseases and was carried out to help guide the development of an IVM programme to assist the elimination of both diseases.

## Materials and Methods

**Figure 1:**
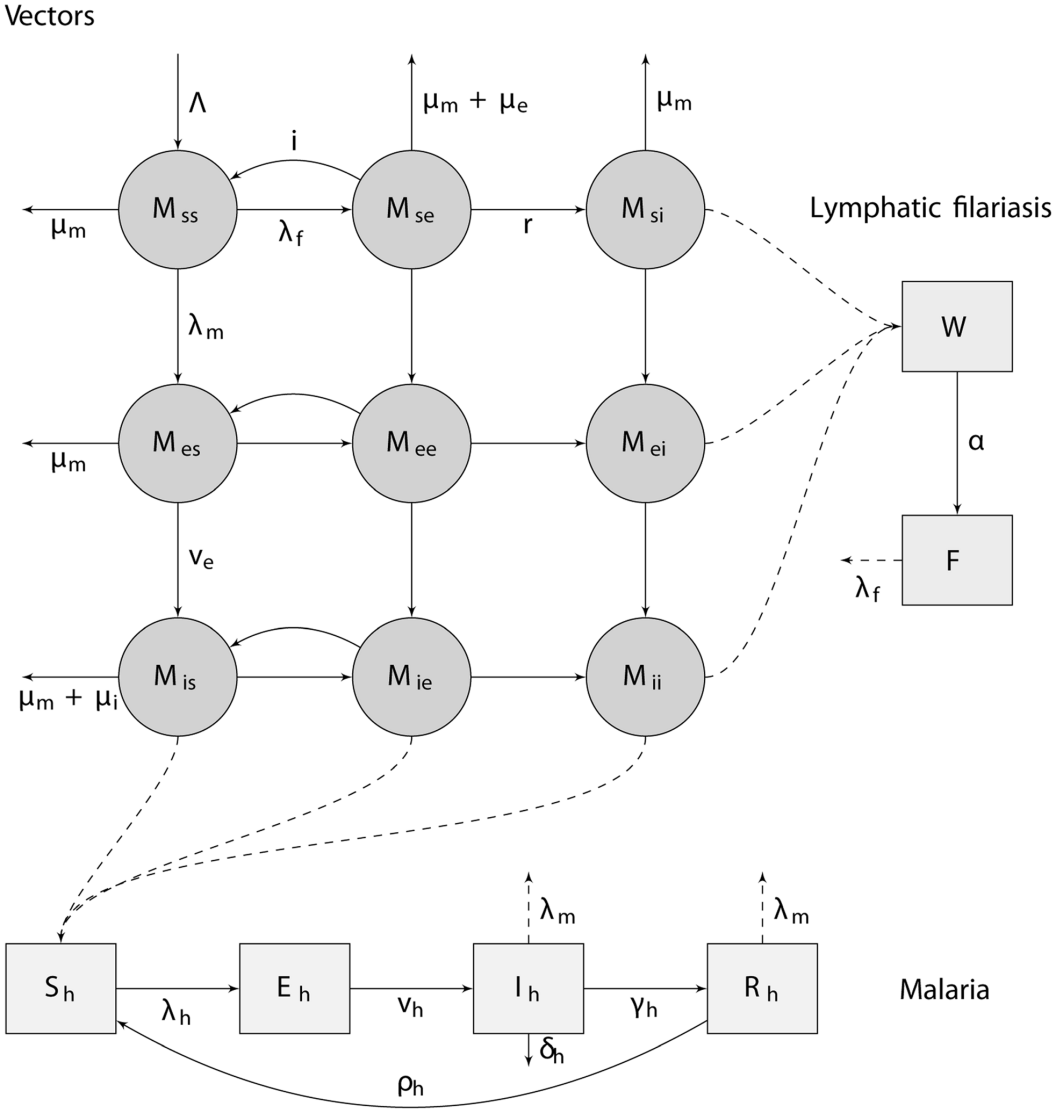
A diagram of the compartments and transitions between them used in the transmission model. In humans, malaria infection is modelled using a susceptible-exposed-infective-recovered prevalence-based system, while for lymphatic filariasis the mean worm and microfilariae burden are tracked. A description of the individual compartments is given in Table 1, and rate parameters are described in Table 2. Interaction between the parasites occurs in the vector due to induced-mortality. All mosquitoes have a constant background mortality rate of μ_m_; mosquitoes that are infectious with malaria have an additional mortality rate, μ_i_; and mosquitoes that are exposed to LF have an additional mortality rate, μ_e_.

We model both malaria and LF as a system of ordinary differential equations, representing mean filarial worm and microfilariae burdens in humans based on parasite burden helminth models [25], and proportions of the human population that is susceptible, infected but not yet infective, infective, or immune to disease for malaria, based on extensions of the Ross Macdonald model [26], with no interaction of parasites in humans. We assume susceptible-exposed-infectious prevalence dynamics for both diseases in mosquitoes with the possibility of co-infection. For LF infection in mosquitoes, since this entails modelling the prevalence of infection rather than the mean larval burden of each mosquito, we have made the assumption of strong density-dependence in the parasite action on the vector (as in some models for schistosomes [27]). All state variables of the ordinary differential equations are shown in Table 1. A diagrammatic overview of the model is given in Fig. 1. Adult filarial worm and microfilariae burdens in humans are modelled in a similar way as other deterministic filariasis models [28], [29], although age-dependence in humans and potential effects of immunity on worm establishment (simplifications shared with [22] and [16], respectively) are ignored.

**Table 1:** Description of state variables.

State variable	Description
S_h_	Number of humans who are not infected with malaria but may get infected if bitten by infectious mosquitoes.
E_h_	Number of humans that are infected with malaria, but not yet infectious
I_h_	Number of humans that are fully infectious for malaria
R_h_	Number of humans in the recovered state who are immune to clinical disease but still harbour low levels of parasites and are still infectious to mosquitoes (albeit at a lower probability)
W	Mean *W. bancrofti* adult worm burden in the human population
F	Mean microfilariae burden in the human population
M_ss_	Number of mosquitoes uninfected with either parasite
M_se_	Number of mosquitoes exposed to *W. bancrofti*, but not infected with *P. falciparum*
M_si_	Number of mosquitoes with infectious stage *W. bancrofti* larvae, but not infected with *P. falciparum*
M_es_	Number of mosquitoes exposed to *P. falciparum*, but not infected with *W. bancrofti*
M_ee_	Number of mosquitoes exposed to *P. falciparum* and *W. bancrofti*
M_ei_	Number of mosquitoes exposed to *P. falciparum*, and infectious for *W. bancrofti*
M_is_	Number of mosquitoes with *P. falciparum* sporozoites, but not infected with *W. bancrofti*
M_ie_	Number of mosquitoes with *P. falciparum* sporozoites, and exposed to *W. bancrofti*
M_ii_	Number of mosquitoes infectious for both *P. falciparum* and *W. bancrofti*

We use a susceptible, exposed, infective, resistant and susceptible (SEIRS) compartmental model for malaria, following Chitnis et al [30], ignoring human migration and assuming a constant per capita density independent death rate.

We use the recovered class, *R_h_,* to model immunity to malaria in humans, where people harbour low levels of parasite in their blood that are often undetectable but still allow a lower probability of transmission to mosquitoes. Although in reality, humans frequently move between patent and sub-patent parasitaemia, and the duration of infection may be dependent on past exposure, we make the simplifying assumption here that the recovered stage has a fixed duration. Equations and further details are provided in the S1 Supporting Information.

**Figure 2:**
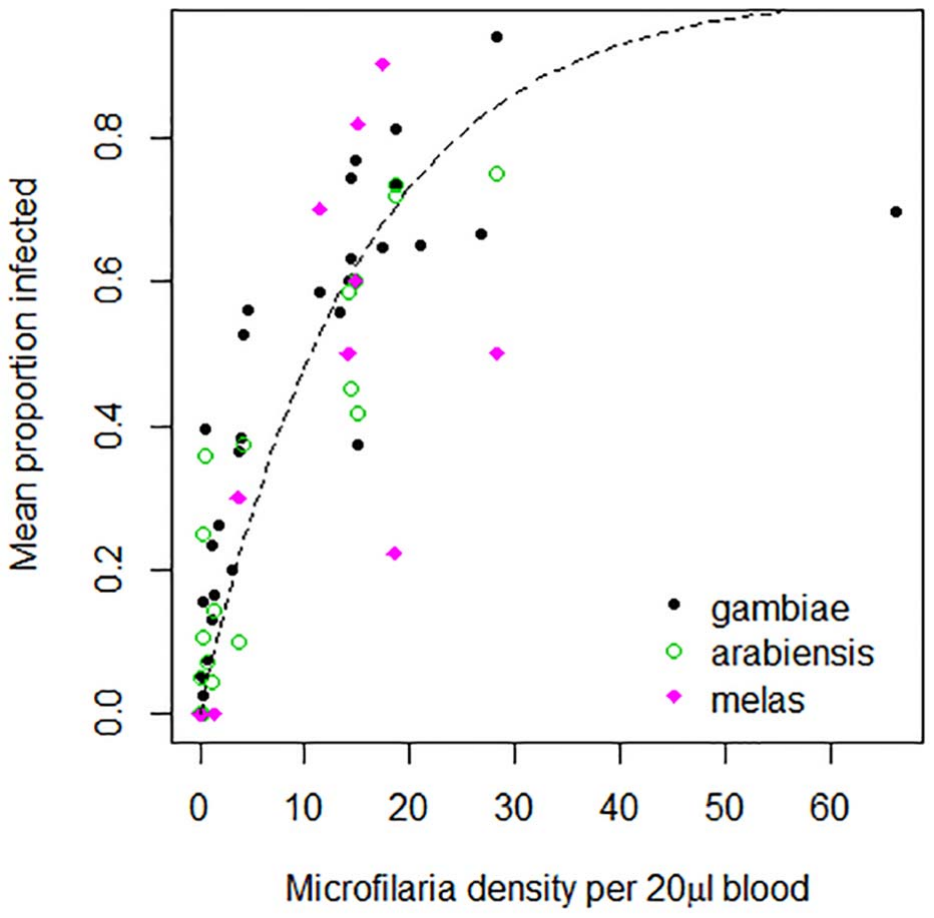
The probability of a mosquito being infected with LF per bite on an infectious human, as a function of the microfilariae density in humans, from [32] and [34]; with best fitting curve using non-linear least squares.

The number of microfilariae ingested by mosquitoes depends on the density of microfilariae in the blood meal [31]. For our prevalence based model, we fitted an exponential curve to data on the probability of ingesting microfilariae for *An. gambiae, An. arabiensis* and *An. melas* [32], [33], [34] (Fig. 2). The probability of a mosquito ingesting microfilariae when biting a human with microfilariae is given by the following equation: 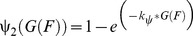
(1)


Where *G* reflects the mean microfilariae burden among infected humans,
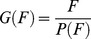
(2)and *P(F)* is the prevalence of infection depending on the mean microfilariae burden. We approximate a negative binomial relation between microfilariae burden and prevalence of infection as in [28] with
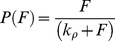
(3)


The force of infection on mosquitoes is:
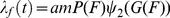
(4)where *a* is the number of bites per mosquito per day and m is the proportion of ingested microfilariae that passes the midgut barrier (see the supplementary material). The force of infection for malaria of human to mosquito, *λ_m_*, is:
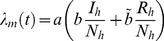
(5)where *b* is the likelihood of a mosquito becoming infected when feeding on an infective human, and 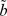 the likelihood when feeding on an immune human.

### Effects of parasitic-infection on mosquito survival

Interactions between parasites and vectors are multi-faceted and likely depend on the co-evolutionary history of the association. Filarial larvae can exert a number of costs on the mosquito, including damage inflicted while crossing the midgut, while developing in the thoracic musculature, or due to breakage of the labium [35], or a metabolic cost associated with an immune response to infection [36], with high mortality typically associated with high numbers of larvae. Relatively few studies have looked into this matter for the natural pairing of *Anopheles* spp. and *W. bancrofti* infection and a meta-analysis did not support the notion of density-dependent mortality for this pairing [37]. Although recently in *An. farauti* density-dependent mortality due to filarial infection was observed [38] and field data has also suggested this for *An. gambiae s.l.* and *An. funestus* [39]. The effects of infection with *Plasmodium* on *Anopheles* survival is likewise equivocal, with evidence suggesting it occurs mostly based on experimental systems consisting of mosquito-*Plasmodium* species combinations that do not occur in nature, such as *An. stephensi* infected with *P. berghei* [40], [41]. This induced mortality was most likely be due to the sporozoite stage [40], [42], but has also been linked to the oocyst burden [43]. We make the assumption that additional mortality due to harbouring sporozoites and filarial larvae occurs so additional malaria mortality acts only on infectious mosquitoes and additional LF mortality acts only on exposed mosquitoes. Since we use a prevalence- rather than intensity-based model for infection with LF, we assume a constant level of mortality. Putatively, susceptibility to co-infection could be likelier as infection with one parasite could weaken the mosquito's innate defences against subsequent infection, and survival of co-infected mosquitoes could be decreased further, but we do not model this effect here. Evidence for the first comes from field studies finding higher than expected proportions of mosquitoes carrying co-infections [44], [45], but the opposite has also been reported [46], and laboratory experiments suggest filarial infection may reduce development of *Plasmodium* in vectors [47] so we ignore this effect in our model. In terms of mortality, we assume the parasite-induced mortality acts additively. Further details on parameter values used for mosquito-filaria interactions are provided in S1 Supporting Information.

### Parameter uncertainty, density dependence, and environmental variation

To investigate the robustness of our baseline analyses we performed simulations allowing for model parameter uncertainty, environmental variation in the form of seasonality, and density-dependent larval mosquito survivorship.

To explore the impact of vector control methods on *P. falciparum* and *W. bancrofti* prevalence we ran a series of 500 simulations. The parameter sets used were drawn using Latin hypercube sampling from uniform distributions of parameter values with ranges as specified in Table 2.

**Table 2:** Parameter descriptions and values used.

Parameter	Description	Baseline value	Range	Unit	Source
ψ_h_	constant birth rate of humans	0.0000421	-	d^−1^	-
ρ_h_	waning of malaria immunity in humans	0.00055	5.5×10−4–2.7×10−3	d^−1^	[54]
a	mosquito biting rate on humans	0.33	0.2–0.5	d^−1^	[67]
c	likelihood of malaria infection in humans following an infectious bite	0.022	0.011–0.033		[54]
μ_h_	human death rate	0.0000421	-	d^−1^	-
v_h_	human rate of progression from the exposed to the infected state for malaria	0.1	0.067–0.20	d^−1^	[54]
γ_h_	human recovery rate from malaria	0.0035	0.0017–0.007	d^−1^	[54]
δ_h_	malaria-induced mortality rate for humans	0.00009	0−1.8×10−5	d^−1^	[54]
b	likelihood of a bite on an infected human leading to malaria infection in mosquitoes	0.097	0.08–0.114		[18]
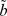	likelihood of a bite on a recovered human leading to malaria infection in mosquitoes	0.0097	0.008–0.0114		[54]
ψ_1_	likelihood of an infectious bite leading to establishment of a worm in humans	0.000036	3.6×10−5–8.8×10−5		[16], [19], [28]
L_3_	mean number of 3^rd^ instars carried by infective mosquitoes	1.48	1–1.98		[34], [68], [69]
μ_w_	death rate of adult worms in humans	0.00034	5.48×10−4–2.74×10−4	d^−1^	[28]
α	worm fecundity in humans	0.0667	6.67×10−3–6.67×10−2-	d^−1^	[28], [59]
μ_f_	death rate of microfilariae in humans	0.0033	0.002–0.004	d^−1^	[28]
Λ	emergence of mosquitoes	8000/16000	varies seasonally	d^−1^	-
ψ_2_(F)	likelihood of *W.b.* infection in mosquitoes when feeding on a microfilaremic host	varies	-		
r	rate of *W.b.* larval development within mosquitoes	0.083	0.0714–0.1	d^−1^	[70]
v_e_	reciprocal of the extrinsic incubation period for malaria	0.0909	0.0714–0.1	d^−1^	[54]
μ_m_	death rate of mosquitoes	0.1	0.05–0.15	d^−1^	[56]
μ_e_	*W.b*.-induced additional mortality of mosquitoes	0.059	0.009–0.109	d^−1^	S1 Supporting Information
μ_i_	*P.f*.-induced additional mortality of mosquitoes	0.03	0–0.06	d^−1^	estimate
m	probability of ingested microfilariae passing the midgut in mosquitoes	0.636	0.4–0.87		S1 Supporting Information
i	loss of *W.b*.-infection in mosquitoes due to immune response	0.37	0.13–0.61	d^−1^	S1 Supporting Information
k_ρ_	factor used to relate microfilariae burden to prevalence	8.9	-		[28]
k_ψ_	constant in microfilariae uptake function	0.06539	0.0554–0.0772		[32], [34]

Here, mosquito immatures were assumed to be subject to density-dependent mortality, based on the formula of Dye [48], so that the daily emergence of female mosquitoes is given by:
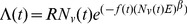
(6)where E represents the number of female eggs oviposited on average by a mosquito per day, R the number of offspring that would emerge were only density-independent mortality to operate, and f(t) and β modify the strength of density-dependent immature mortality.

The effect of seasonality on mosquito population dynamics was assumed to operate through the effect of rainfall patterns on the number and size of available larval development sites. We approximated this effect by making the strength of density-dependent mortality, f(t) vary following a sinusoidal pattern:
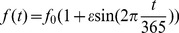
(7)where f_0_ is the baseline value of f(t) and ε regulates the strength of seasonal variation.

## Results

### Basic reproductive numbers for malaria and lymphatic filariasis

The basic reproductive number, or ratio, R_0_, estimates the number of secondary infections that result from the infectious duration of a single case in a fully susceptible population and thus it provides a basis for judging whether a disease will thrive or be eliminated. When R_0_ is less than or equal to 1 the disease will be eliminated, whilst if it is greater than 1, the disease will survive. We derive expressions for the basic reproductive numbers of malaria and LF (where R_0_ represents the number of adult filarial worms arising from one adult filarial worm in the absence of density dependent regulation) in the absence and in the presence of the other pathogen, using a next-generation matrix approach [49], [50]. The derivations are provided in the supplementary material. The resulting expression for malaria in the absence of LF is:
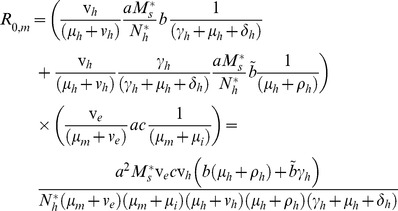
(8)


Where the first term represents the probability of a person in the latent stage progressing to the infective stage rather than leaving the compartment through dying, multiplied by the average amount of time spent in the infective stage, multiplied by the number of mosquito bites on that person per day that result in infection of a vector. The second term stands for the probability that a latent human passes to the immune stage (through the infective stage), the average amount of time spent in the immune or recovered stage, multiplied by the number of bites received per day that result in vector infection. The third term is then the probability of an infected vector progressing to the infective stage times the bites per day that result in infection in humans, multiplied by the average amount of time spent in the infective stage.

While for LF in the absence of malaria we obtain:
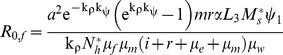
(9)


Which, following a similar logic, consists of terms representing the fecundity of an adult worm and the mean lifespan of adult worms and microfilariae, multiplied by the number of mosquito bites on the population that result in establisment of infection in the vector, and terms for the probability that an infected mosquito progresses to the infective stage, the average duration of the infective stage, and the number of infective larvae that are delivered per day per human that reach maturity. Here 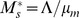 and 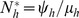 are the equilibrium values of the mosquito population and human population, respectively, in the absence of both diseases.

For malaria in the presence of filariasis and for filariasis in the presence of malaria the basic reproductive numbers are given by large expressions (see the supplement), but we note that these reduce to the expressions similar to the equations above when parasite-induced mortality due to the other parasite is ignored.

### Equilibrium values

**Figure 3:**
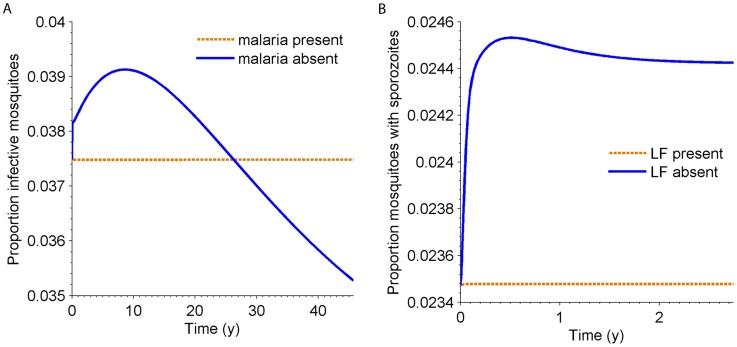
A) Proportion of mosquitoes infectious with LF when malaria is removed from the co-endemic equilibrium; B) the proportion of mosquitoes infectious with malaria when LF is removed from the co-endemic equilibrium. The negative effect of LF on malaria is due to the increased mortality associated with harbouring *W. bancrofti* larvae, while the effect of malaria on LF is a balance between the additional death rate on humans, increasing the mosquito∶human ratio, and the *Plasmodium*-induced mosquito mortality.

Potential positive or negative side effects associated with disease control methods that target a single parasite only (for instance, due to treatment of humans) can be investigated by simulating the artificial removal of either parasite from the co-endemic equilibrium state. The effects on the proportion of infectious mosquitoes of either disease (i.e. harbouring sporozoites or third-stage larvae) after setting all human and mosquito infections to zero for the other disease are shown (Fig. 3). When malaria is removed, LF transmission intensity is expected to initially increase, before decreasing, eventually to a slightly lower equilibrium. The initial rise in the proportion of infectious mosquitoes can be ascribed to the removal of *Plasmodium*-induced mosquito mortality. Over a much longer period this is balanced by the adjustment in human population size following the removal of malaria-induced human deaths. The implications or removing LF on malaria transmission, based on our model, are more straightforward as transmission will increase in intensity due to the absence of filarial-induced vector mortality.

### Sensitivity analysis of R_0_ to transmission-related parameters

**Figure 4:**
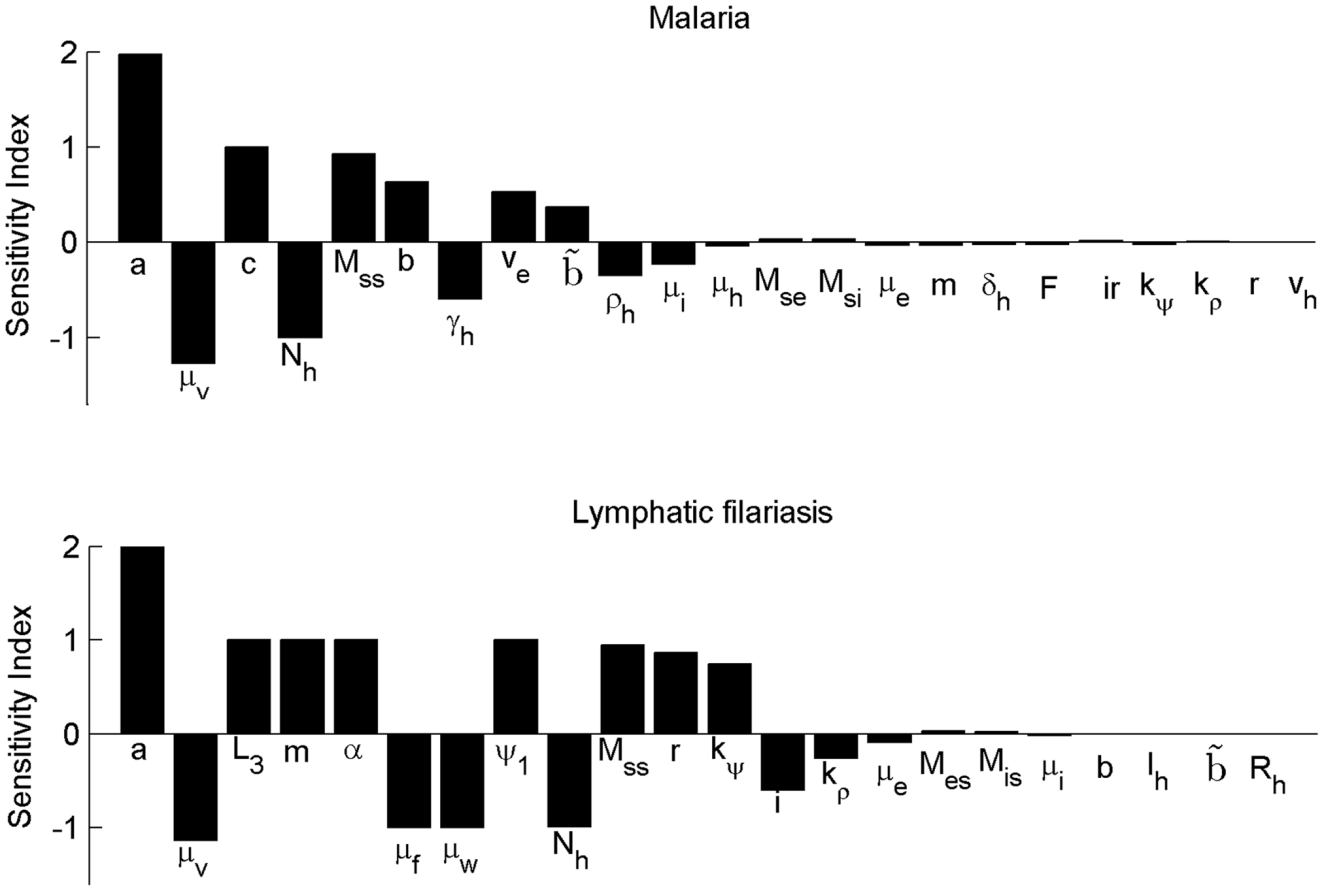
Local normalized sensitivity analysis of R_0_ to model parameters for malaria and lymphatic filariasis in the presence of the other disease for parameter values given in Table 2. Endemic equilibrium values for mosquito populations for the other disease are calculated numerically.</emph>

To determine the key variables that drive transmission of LF and malaria in the presence of the other disease a local sensitivity analysis of R_0_ was performed by calculating the normalized forward sensitivity indices [51], equivalent to the concept of elasticity [52], [53], as:
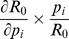
(10)where *p_i_* is any parameter of the model. Such an analysis can help identify which parameters influence R_0_ most strongly and therefore make appealing targets for disease management [52]. If high impact variables are shared between the two diseases, these should be the focus of integrated strategies. We follow the methodology as described in [54] and evaluate the sensitivity index of R_0_ for malaria at the malaria-free endemic equilibrium point for LF, and *vice versa.* The values for each of the parameters that constitute the expressions of R_0_ are given in Fig. 4. For both diseases, the most important parameter is the biting rate, *a*, followed by the base mosquito mortality, μ*_m_*. Notable is that the parasite-induced mortality (μ*_i_* for malaria, μ*_e_* for filariasis) has only a weak impact, while parameters related to transmission of the other parasite, including parasite-induced mortality, have a relatively minimal impact. This suggests that although removal of one parasite could (temporarily) increase transmission of the other parasite, such an effect should be overshadowed if interventions simultaneously target a shared parameter, such as the biting rate, vector mortality or density.

### Impact of vector control

We investigate the impact of vector control by calculating the basic reproductive number of malaria and LF over a range of coverage levels (indicated by φ), and by numerical simulations. Both LLINs and larval control are considered due to their targeting of different vector-related parameters: the mosquito biting rate and feeding-related mortality, and the emergence rate and resulting mosquito density. Larval control is here modelled in a simplified manner and assumed to have a linear effect on the daily emergence of mosquitoes, Λ, so that a 90% coverage of breeding sites is assumed to reduce emergence of adults by 90% (but note that a recent paper suggests that treating 50% of breeding sites was associated with a 90% reduction in adult mosquitoes [55]). The equilibrium number of susceptible mosquitoes then becomes Λ(1-φ)/μ_m_. We note here that in certain settings such as urban areas, high coverage of larval breeding sites may be possible, but in many areas it may be difficult to achieve such high coverage.

LLINs are investigated using the formulae derived by Le Menach et al [56] for the feeding cycle duration and probability of survival under varying LLIN coverage levels, and substituting these equations in our expressions for R_0_ (further details provided in the supplement). We make the simplifying assumption that mosquitoes are fully anthropophilic, but this can be relaxed if species-specific differences in feeding behaviour are of interest.

**Figure 5:**
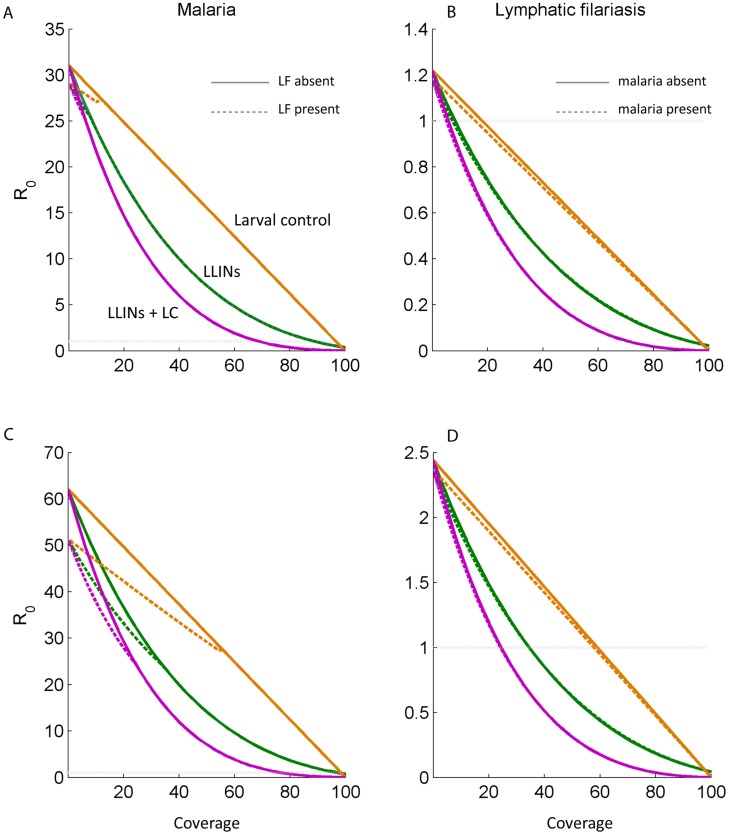
Values of the basic reproductive number, R_0_, for malaria (panels A and C) and lymphatic filariasis (panels B and D) in the presence (dashed lines) and absence (solid lines) of the other parasite, over different coverage levels of interventions, φ. The interventions considered were larval control, insecticide-treated nets, and a combination of both. The results for a mosquito density of 80 per human are depicted on a linear scale in panels A–B. Panels C–D depict the results for a mosquito density of 160 per human.

The impact of LLINs and larval control over a range of 0–100% coverage on the basic reproductive number of malaria in the absence and presence of LF, and of LF in the presence and absence of malaria, is presented (Fig. 5). In both cases, the effect of an interaction with the other parasite is very small. For malaria this is particularly the case, because the range of coverage levels where filariasis persists is very narrow and above that level the expressions of R_0_ are equivalent. This is the case because our model, evaluated at the parameter values specified, predicts a very low R_0_ of filariasis even in the absence of interventions, and a very low level of vector control (6% LLIN coverage or 20% coverage with larval control) is sufficient to reduce this below one. The R_0_ for malaria is greater and requires coverage of 90% of bed nets, 100% of larval control by itself, or 70% coverage of both ITNs and larval control. At a higher mosquito density (160,000), 36% coverage of LLINs, 58% of larval control, or 26% of the interventions applied together, are sufficient to reduce the basic reproductive rate of LF to below one, while 78% of LLINs combined with larval control is now required for malaria, while 100% coverage of either on its own is required to eliminate malaria transmission.

**Figure 6:**
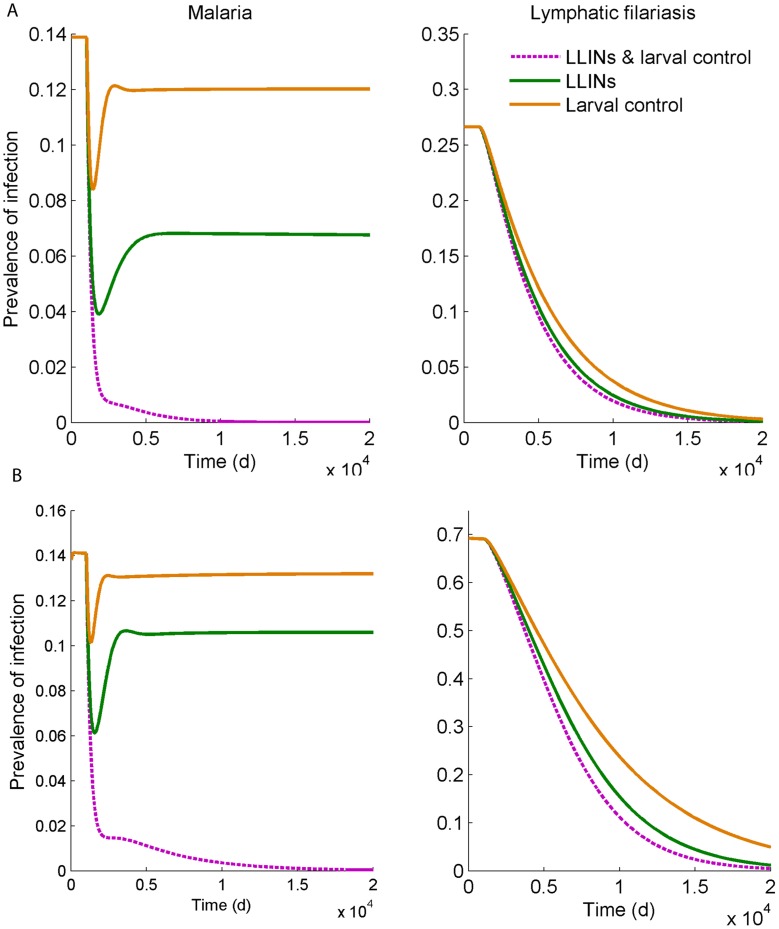
Simulations of the effect of vector control interventions (larval control, insecticide-treated nets, or both) on the prevalence of patent infection of malaria (left) and lymphatic filariasis (right) over time, assuming the intervention starts at day 1000 at a level of coverage, φ, of 80%, when the mosquito density was 80 per person (A) or 160 per person (B). Patent prevalence is defined as the proportion of infectious humans.

**Figure 7:**
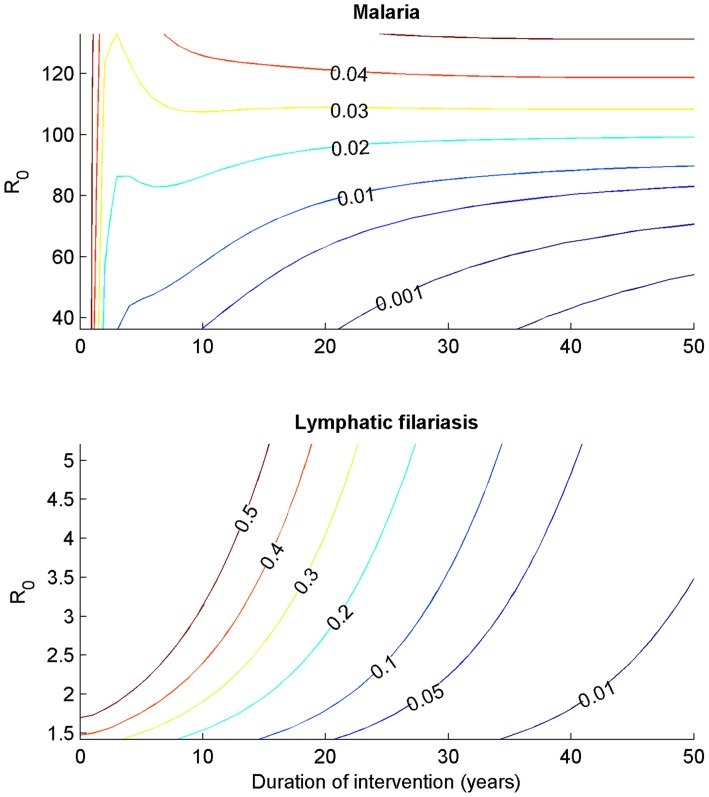
Contour plots of prevalence of infection with *P. falciparum* and *W. bancrofti* (labeled, colored lines) for different levels of pre-intervention R_0_ (generated by varying the mosquito density from 80–330 thousand). The intervention simulated was the combination of long-lasting insecticidal nets and larval control at 80% coverage. For malaria, particularly at the lower ranges of R_0_, the initial decline is rapid, whereas the decline for Lf is slower due to the longevity of the adult worms in humans.

**Figure 8:**
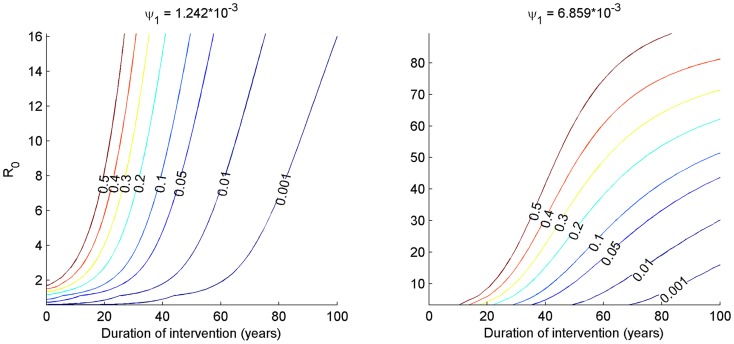
Contour plots of LF prevalence over time with 80% coverage of LLINs and larval source management, over a range of initial R_0_ values corresponding to monthly biting rates of approximately 100–3000 per person. The plots are produced based on two greater values for the efficiency of transmission, ψ_1_. In general, R_0_ reaches higher values as mosquito densities are increased and the duration interventions have to remain in place are longer as ψ_1_ increases. At the greatest efficiency (right panel) vector control does not lead to elimination at high mosquito densities.

**Figure 9:**
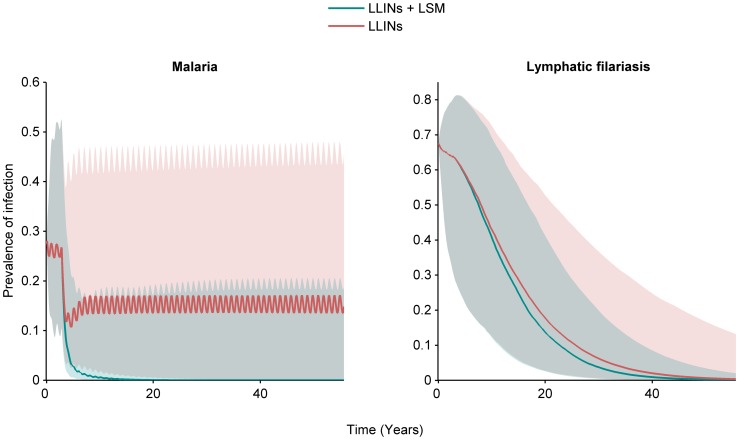
Median (solid lines) and 95^th^ percentile range (shaded areas) of 500 simulations of the effect of vector control interventions (long-lasting insecticidal nets, or LLINs & larval control) on the prevalence of patent infection of malaria (left) and lymphatic filariasis (right) over time, assuming the intervention starts at day 1000 at a level of coverage, φ, of 80%. Simulations reflect the impact of uncertainty in model parameter values, and seasonal variation in density-dependent immature mosquito mortality. Patent prevalence is defined as the proportion of infectious humans.

Fig. 6 shows the effects of vector control measures when both diseases are present when simulated over time, in this case at a level of coverage, φ, of 80%, for mosquito population sizes of 80,000 and 160,000. The results are in line with those of Fig. 5, and show with that larval control or bed nets by themselves, malaria prevalence would reach a lower equilibrium, whereas prevalence is reduced to zero over time when LLINs are combined with larval control. For LF all interventions are sufficient to eliminate the disease, although this reduction takes place over a longer period. For instance, the time that interventions have to be in place to reduce the prevalence of patent infection to one half that of the initial, equilibrium level of prevalence was 218 days for malaria compared to nearly 8 years (2,884 days) for LF, when LLINs are combined with larval control, for the lower mosquito population size. To achieve a 32-fold reduction takes 10 years (3,552 days) for malaria, and 32 years (11,554 days) for LF, under those same conditions. The impact of a wider range of R_0_, simulated by varying the mosquito density between 80000 and 330000 on the prevalence of infection over time when LLINs and larval control are both employed at 80% coverage, is shown (Fig. 7). As the mosquito density increases far enough, malaria again reaches a new equilibrium, whereas for the parameter values used, LF will be eliminated at this level of vector control even at high mosquito densities. The robustness of this outcome to varying a key parameter regarding the efficiency of establishment of new adult filarial worms is provided (Fig. 8), while the impact of uncertainty in parameter values overall as well as environmental variability in the form of seasonally varying density-dependent immature mosquito mortality is explored in Fig. 9. For LF, the 95^th^ percentile range decreased to zero over time for both LLINs used alone as well as in combination with larval control, with only the rate of decline being affected. There was more variability in the response of malaria, although the overall pattern corresponded to that of our baseline investigation (Fig. 6). When both LLINs and larval control were employed, 84% of simulations had reached a prevalence <1% at the end of the projected period. When only LLINs were used, only 5% of simulations had reached that level.

## Discussion

The principal findings of this analysis were that the basic reproductive number, R_0_, of both LF and malaria can be brought to below one by controlling their anopheline vectors. The value of R_0_ of LF was found to be lower than the R_0_ of malaria, and consequently controlling LF required a lower level of population coverage with LLINs and larval source management than did controlling malaria. However, the time over which the prevalence of infection is reduced is longer for LF than for malaria.

The difference between the basic reproductive numbers and consequently the critical densities of mosquitoes of malaria and LF are striking, though not unexpected. Care does have to be taken with the comparison, because while both indicate threshold criteria, there is a difference in interpretation where for malaria, R_0_ represents secondary cases of humans and for LF the number of adult worms arising from one worm [50]. A question that arises is whether we see in the field that the two diseases co-occur mostly in areas with high malaria transmission, as predicted here. It does raise the question whether the low value of R_0_ of filariasis in combination with the mosquito density required to maintain transmission that we found in our analysis is representative of field situations.

Threshold biting rates between 200–300 per month were reported in a theoretical study for Anopheles spp., and as low as 9 bites per month for LF transmitted by Culex spp [29]. Stolk et al. [16] reported a threshold in their model at approximately 400 bites per person per month. Our prediction was approximately 675 bites per month, and the critical density for filariasis predicted by our model of 67 mosquitoes per human is likewise higher than the value of 20 calculated by Webber [17], while in a later field study a density of 60 was found [57]. Our values appear slightly higher, perhaps as a result of the additional vector mortality due to infection included here.

The main difference in the values of R_0_ between malaria and LF clearly depends on the parameters that are not shared between equations (6) and (7). As the filarial worms are long-lived and fecund, and the extrinsic incubation periods comparable between the parasites, this most likely then hinges on either parameters associated with uptake and establishment of filarial larvae in the mosquito (k_ψ_, i, m), or, more likely with the (in-)efficiency of establishment of (mated, female) adult worms from infective bites (ψ_1_). A central criticism of our work is that this inefficiency of LF transmission stems from only one field study in Myanmar (formerly Burma) and an estimate from one prior modelling study [28] and clearly this needs empirical confirming in other parts of the world, including sub-Saharan Africa. This parameter, ψ_1_, represents an amalgamation of a number of factors, such as the probability that an infective filarial larva egresses from the labium during a blood meal, the probability that the larva successfully enters the puncture wound created by the blood-feeding mosquito, and the probability that the larvae survives, develops and mates within the human. The second, the probability that an infective larvae exposed on the human skin in a drop of haemolymph survives and enters the blood stream has been shown to depend on the relative humidity and rate of evaporation [58], and may thus show considerable geographic or seasonal variation.

The values for this parameter used in other recent LF models are also variable, ranging from 2.4–3.4 times higher than our value [16], [22] to ca. 18 [59] and 700 [29] times greater. Note that a direct comparison of these values is complicated by the inclusion of mating functions and particularly immunity affecting worm establishment in some of these more complex models. The range for this value used in our global uncertainty analysis was therefore based on values used in additional models without immunity (Fig. 9), though a wider range was considered in a one-way sensitivity analysis (Fig. 8). Our conclusions are robust to parameter uncertainty overall, as well as the inclusion of seasonal mosquito population dynamics (Fig. 9). The implications of seasonality appear more pronounced for malaria than they do for lymphatic filariasis, likely as a result of the shorter duration of Plasmodium infection in humans and indirect effects on acquired immunity. Our predicted outcomes are robust to changes in mosquito abundance (Fig. 7) as well as the inefficiency of Wuchereria transmission if we increase this parameter value (ψ_1_) 3.4-fold, but if increased 18-fold (as in certain models where immunity is assumed to negatively affect worm establishment) then at higher mosquito densities, 80% coverage of both LLINs and larval source management are predicted to lead to a lower equilibrium prevalence of LF rather than elimination, as is also the case for malaria (Fig. 8). This highlights the need for further model fitting and, given the general paucity of information on the impact of LF on vectors in the wild, the need to collect more field data to help improve the models. However, we note that the intention of the current analysis was not to provide a predictive model, but rather a general model including parasite-vector interactions of malaria and LF, to gain insight into how to integrate vector control for these two diseases. The use of simplifying assumptions is not restricted to the filariasis side of the model. The current malaria model, based on extensions to the Ross-Macdonald model, includes a class of recovered individuals with a lower infectiousness, which is meant to portray both the confounding effects of superinfections resulting in a skewed distribution of infection duration and the effects of acquired immunity in reducing parasite densities. Other models of malaria have included more realistic and complex systems of ordinary differential equations [60] or have used stochastic individual based models [61], [62], [63], and as typical in modelling, depending on the balance between analysis and simulations desired, a higher or lower degree of realism can be adapted. In the case of parasite-vector interactions greater realism could come from modelling these processes (mortality and immunity) as functions of parasite density. Since we were most interested in the potential negative side-effects of controlling only one disease and how this could be mitigated by vector control, we included only mortality induced by parasites, but a more realistic model may have to additionally consider other behavioural modifications, such as an increased biting rate.

A further caveat to this model is that homogeneous mixing of vectors and hosts is assumed, but in reality fine spatial variation, for instance due to microclimatic differences or proximity to mosquito breeding sites, will exist and potentially allow for transmission hotspots where R_0_ will be elevated and transmission harder to interrupt [64], [65]. To gain insight into interactions between the two diseases at such a scale, modifications will be required to the model structure to take heterogeneous exposure to mosquito bites into account. In addition to the differences in R_0_ between LF and malaria, the difference in response times to vector control interventions was notable, with LF requiring longer durations of interventions to reach low levels of prevalence due to the longevity of the adult filarial worms. For malaria there is an initial fast drop-off as the proportion of humans in the infective compartment is rapidly reduced, followed by a slower decline as individuals remaining in the less infectious but longer-lasting recovered compartment continue to contribute to transmission. Note however, that we have focused solely on vector control and not included the use of antihelminthic or antimalarial drugs in this study. Worm burdens will fall more rapidly when diethylcarbamazine, ivermectin, or albendazole can be administered to the population and should therefore remain a priority, even in areas that have been subject to malaria-vector control for many years.

An additional consideration is that the modelling approach we used likely overestimates the required duration that interventions have to remain in place for LF, because in our model mortality of filarial worms follows an exponential function and transmission can resurge with a small fraction of infected cases. A model that includes stochasticity, a more suitable distribution for worm lifespan than an exponential one (e.g., a Gompertz function), as well as a mating function for filarial worms, would be more suitable for accurately determining the duration of programs needed for elimination. In our simulations, the R_0_ of LF required only very modest levels of vector control coverage to be brought below one. A consequence of this is that larval control, which by itself was not sufficient to control malaria in our simulations, was sufficiently effective to halt filariasis transmission. However, even these low levels of coverage had to be maintained over a long period. Larval control, for instance, had to be maintained for nearly 10 years to halve the initial prevalence of infection, or approximately 41 years to achieve a 32-fold reduction in prevalence. Faced with the rise in pyrethroid-resistance seen with An. gambiae s.l. and the relatively poor ability of nets to kill culicine mosquitoes [66], applying larval control should be considered as a supplementary measure for filariasis control in addition to LLINs. Such an approach could be effective across mosquito genera and ecological settings. Larval source management could also supplement LLINs in areas with very high malaria transmission where use of LLINs at high levels of coverage are insufficient to severely dampen transmission. This would be particularly attractive if larval control activities can be maintained over longer periods (30–40 years in these simulations) by communities than the average lifespan of LLINs.

Our combined malaria and LF model takes into account that both *Plasmodium* and *Wuchereria* parasites may have adverse effects on the survival of their vectors, agrees with previous investigations that interventions that target only one of the parasites may have negative, unintended consequences on transmission of the other parasite [20], [21], [22]. However, the outcome of our sensitivity analysis suggests that transmission of both malaria and LF are most strongly impacted by perturbations of the mosquito biting rate, and vector mortality. Perturbation of parameters related to the other disease has a relatively minor impact, suggesting that any putative negative consequences of disease control will be overshadowed by the implications of vector control measures. That LLINs, which affect the biting rate and vector mortality, have a stronger impact on the basic reproductive number than larval control, which (in our simplified model without a full model of mosquito population dynamics) only affects mosquito density in a linear fashion, is thus in agreement with the sensitivity indices. However, it should be appreciated that in our model the impact of larval control is likely to be underestimated [55]. This lends further support to the notion of integrating the rollout of LLINs into LF drug administration campaigns [2], [14]. Based on the sensitivity analysis, indoor residual spraying (IRS) would be expected to have an impact on LF transmission (see also [11]) comparable to LLINs, but whether it should be considered as an alternative or in addition to LLINs may depend more on issues related to the cost-effectiveness of performing spray rounds for a sufficient amount of time needed to eliminate LF (i.e. at least 4–8 years).

## Conclusion

This analysis confirms that the massive roll-out of LLINs for malaria control will have additional impact on the transmission and control of LF. Elimination of LF via vector control only is plausible, but likely only feasible in the form of mosquito abatement sustained over many years. The synergies that come from attacking two diseases with the same interventions should be exploited to a greater extent in elimination programmes. This is particularly relevant in West Africa where drug treatment against LF cannot be administered in areas endemic for loaiasis. LLINs and, where applicable, larval source management should be used for the control of malaria and LF in areas where both diseases are transmitted by the same vector.

## Supporting Information

S1 Supporting Information Detailed descriptions of the model equations (section I); *Wuchereria-Anopheles* interactions included in the model (section II); the formulae used to represent the effects of long-lasting insecticidal nets (section III); and the basic reproductive numbers for malaria and lymphatic filariasis (section IV).
(DOCX)
