# Supplementary material for: How Effective is Integrated Vector Management Against Malaria and Lymphatic Filariasis Where the Diseases Are Transmitted by the Same Vector?
Source: PLoS Negl Trop Dis. 2014 Dec 11;8(12):e3393. doi: 10.1371/journal.pntd.0003393 (PMC4263402; doi:10.1371/journal.pntd.0003393)
Supplement: S1 Supporting Information — Detailed descriptions of the model equations (section I); Wuchereria-Anopheles interactions included in the model (section II); the formulae used to represent the effects of long-lasting insecticidal nets (section III); and the basic reproductive numbers for malaria and lymphatic filariasis (section IV). (DOCX) [file pntd.0003393.s001.docx]

How Effective is Integrated Vector Management against Malaria and Lymphatic Filariasis where the Diseases are Transmitted by the Same Vector?

**Supporting Information S1**

Christopher M. Stone, Steve W. Lindsay, Nakul Chitnis

**Section I: Model equations**

The mean worm and microfilariae burden of *Wuchereria bancrofti* in humans in given by the following equations:

|  | $\frac{dW}{dt}= \text{ψ}_{1}a\frac{M_{si}+M_{ei}+M_{ii}}{N_{h}}L_{3}- \mu_{w}W$ | (S1.1) |
| --- | --- | --- |
|  | $\frac{dF}{dt}= \alpha W-\mu_{f}F$ | (S1.2) |

Here W refers to the mean adult worm burden in the human population and F the mean number of microfilariae in 20 μl of human blood; ψ_1_ is a proportion of infective bites that leads to the establishment of a worm; (M_si_ + M_ei_ +M_ii_)/N_h_ is the ratio of LF infective mosquitoes (those with third-stage larvae) to humans and *a* their biting rate on humans (i.e., a combination of host preference and feeding frequency). The value of L_3_ is the mean number of infective larvae carried by infective mosquitoes. The fecundity of female worms is represented by α, and μ_f_ represents the mortality rate of microfilariae. Certain models include a mating function, φ, for dioecious, polygamous worms that are negative binomially distributed, introducing a breakpoint in the mean worm burden below which the infection will die out, although the relevance when infection is highly overdispersed has been questioned [[1](#_ENREF_1)]. For the sake of simplicity we ignore the mating function here.

We use a susceptible, exposed, infective, resistant and susceptible (SEIRS) compartmental model for *Plasmodium falciparum*, following Chitnis et al [[2](#_ENREF_2)], ignoring human migration and assuming a constant per capita density independent death rate:

|  | $\begin{matrix} \frac{dS_{h}}{dt}=\text{ψ}_{h}+\rho_{h}R_{h}-\lambda_{h}\left(t\right)S_{h}-\mu_{h}S_{h} \\ \frac{dE_{h}}{dt}=\lambda_{h}\left(t\right)S_{h}-v_{h}E_{h}-\mu_{h}E_{h} \\ \frac{dI_{h}}{dt}=v_{h}E_{h}-\gamma_{h}I_{h}-\mu_{h}I_{h}-\delta_{h}I_{h} \\ \frac{dR_{h}}{dt}=\gamma_{h}I_{h}-\mu_{h}R_{h}-\rho_{h}R_{h} \end{matrix}$ | (S1.3) |
| --- | --- | --- |

Here *N_h_= S_h_ + E_h_ + I_h_ + R_h_* is the total human population; *ψ_h_* equals births of humans; *ρ_h_* the waning of immunity; and *μ_h_* the base mortality of humans. The reciprocal of the duration of the incubation period, *v_h_*, in humans determines progression from the incubating to the infective stage. Humans leave the infective stage at rates *γ_h_*, the rate of recovery, and *δ_h_*, malaria-induced mortality. The force of infection of mosquitoes to humans, *λ_h_(t)*, is defined as follows:

|  | $\lambda_{h}\left(t\right)= ac\frac{M_{is}+M_{ie}+M_{ii}}{N_{h}}$ | (S1.4) |
| --- | --- | --- |

$\lambda_{h}(t)= ac\frac{M_{\mathrm{is}}+M_{\mathrm{ie}}+M_{\mathrm{ii}}}{N_{h}}$

Where *c* is the likelihood of an infection arising from an infective bite, and the final term represents the number of mosquitoes infective with malaria per human.

Infection with *Wuchereria* and *Plasmodium* in mosquitoes is modelled using susceptible, incubating and infective (SEI) compartments for each parasite:

|  | $\begin{matrix} \frac{dM_{ss}}{dt}= \text{Λ}- \text{λ}_{m}\left(t\right)M_{ss}-\text{λ}_{f}\left(t\right)M_{ss}-\mu_{m}M_{ss}+iM_{se} \\ \frac{dM_{es}}{dt}= \text{λ}_{m}\left(t\right)M_{ss}-v_{e}M_{es}-\text{λ}_{f}\left(t\right)M_{es}-\mu_{m}M_{es}+iM_{ee} \\ \frac{dM_{is}}{dt}= v_{es}M_{es}-\text{λ}_{f}\left(t\right)M_{is}-(\mu_{m}+\mu_{i})M_{is}+iM_{ie} \\ \frac{dM_{se}}{dt}=\text{λ}_{f}\left(t\right)M_{ss}- \text{λ}_{m}\left(t\right)M_{se}-\left(r+\mu_{m}+\mu_{e}+i\right)M_{se} \\ \frac{dM_{ee}}{dt}=\text{λ}_{f}\left(t\right)M_{es}+ \text{λ}_{m}\left(t\right)M_{se}-\left(r+\mu_{m}+\mu_{e}+i\right)M_{ee}-v_{e}M_{ee} \\ \frac{dM_{ie}}{dt}=\text{λ}_{f}\left(t\right)M_{is}+v_{e}M_{ee} -\left(r+\mu_{m}+\mu_{e}+\mu_{i}+i\right)M_{ie} \\ \frac{dM_{si}}{dt}=rM_{se}- \text{λ}_{m}\left(t\right)M_{si}-\mu_{m}M_{si} \\ \frac{dM_{ei}}{dt}=\text{λ}_{m}\left(t\right)M_{si}+rM_{ee}-(\mu_{m}+v_{e})M_{ei} \\ \frac{dM_{ii}}{dt}=v_{e}M_{ei}+rM_{ie}-(\mu_{m}+\mu_{i})M_{ii} \end{matrix}$ | (S1.5) |
| --- | --- | --- |

Here Λ represents a constant rate of newly emerged female mosquitoes with the assumption that emergence is limited by the availability of breeding sites with density dependent larval survival; *λ_m_(t)* is the force of infection from humans to mosquitoes for malaria and *λ_f_(t)* is that for LF (see main article); *μ_m_* represents the base mosquito mortality, *μ_e_* is the additional mortality due to infection with *Wuchereria* (for exposed mosquitoes) and *μ_i_* for *Plasmodium* (for infectious mosquitoes), as described in more detail below. The reciprocal of the development time from ingestion of microfilariae to the appearance of third-stage larvae is given by *r,* while the reciprocal of the extrinsic incubation period for *Plasmodium* is represented by *v_e_*. We assume that mosquitoes with developing filarial larvae can return to the susceptible stage due to an immune response with rate *i*.

**Section II: *Wuchereria*-*Anopheles* interactions**

Mortality due to infection with filariae could, in a prevalence-based model, act on the incubating stage, if mortality is mostly associated with passage through the midgut or as a consequence of an immune response (i.e., a trade-off associated with melanization or upregulation of anti-microbial peptides), or during the infective stage if mortality is mostly associated with migration from the flight muscles and the breakage of the labium and exiting of third-stage larvae [[3](#_ENREF_3)]. Other interactions that have to be considered in such a model are that due to the immune response to infection incubating mosquitoes could potentially return to the susceptible stage. Because larvae do not replicate within the vector, a proportion of mosquitoes harbouring third-stage larvae could lose all larvae and return to the susceptible class (in an experimental system of *Aedes aegypti* and *Brugia pahangi* 86.2% of third-stage larvae escaped bodies of mosquitoes taking a subsequent blood meal [[3](#_ENREF_3)]). To the extent possible, the model should also consider mosquito defences. For instance, a proportion of ingested microfilaria may be shredded by the cibarial or pharyngeal armature. One study found an average of 50.67% of ingested microfilariae to be damaged for *Anopheles gambiae*, *An. arabiensis* and *An. melas* and suggested this effect was density independent [[4](#_ENREF_4)]. An outline of a compartmental mosquito model incorporating all these parameters is given in Fig. S1.

While the greatest insight into density-dependent infection of mosquitoes with filarial parasites and their subsequent development has come from laboratory cohort studies, it is not clear that the infection-related costs incurred under laboratory conditions are representative of those under natural circumstances. Field studies where measures of filarial infection in humans as well as the ratio of infected vs. infective mosquitoes are provided could potentially serve to parameterize these models. If the proportion of ingested microfilariae that develops to the infective stage increases with microfilarial densities, as is suggested for anophelines, it stands to reason that this would be reflected in the proportion of dissected mosquitoes found to be infected or infective. A number of studies have provided both entomological data that distinguishes between infected and infective anophelines, as well as a level of prevalence of infection in humans (though, to our knowledge, not the prevalence and distribution of microfilaraemia intensity among infected humans and detailed entomological data. We thus assume that the relation between mean microfilariae burden in humans and prevalence (equation (3)) holds across these studies, which will introduce some error into these estimations, and are displayed in Fig. S2. This pattern appears to support the notion of facilitation, although we cannot rule out that the pattern is due to other factors such as seasonality or differences in the age-composition of the vector population.

Based on these levels of microfilariae intensity, we estimate the values for the parameters depicted in Fig. S1 using a static model (i.e., assuming a constant microfilariae burden in humans) to minimize the difference between predicted and observed proportions of susceptible, exposed and infective mosquitoes using a non-linear programming tool (Fmincon) in Matlab. The estimated values are shown in Fig. S3. Although some of these parameter estimates are suggestive of density-dependence, at this stage we merely use them to globally inform a simplified model of parasite-vector interactions based on mean values, on which the results are based. Further, we make the simplification that loss of third-stage larvae can be ignored due to the low value estimate and similar function to mosquito mortality and that the induced mortality during the infective stage is negligible compared to that during the incubating stage.

**Figure S1**

**
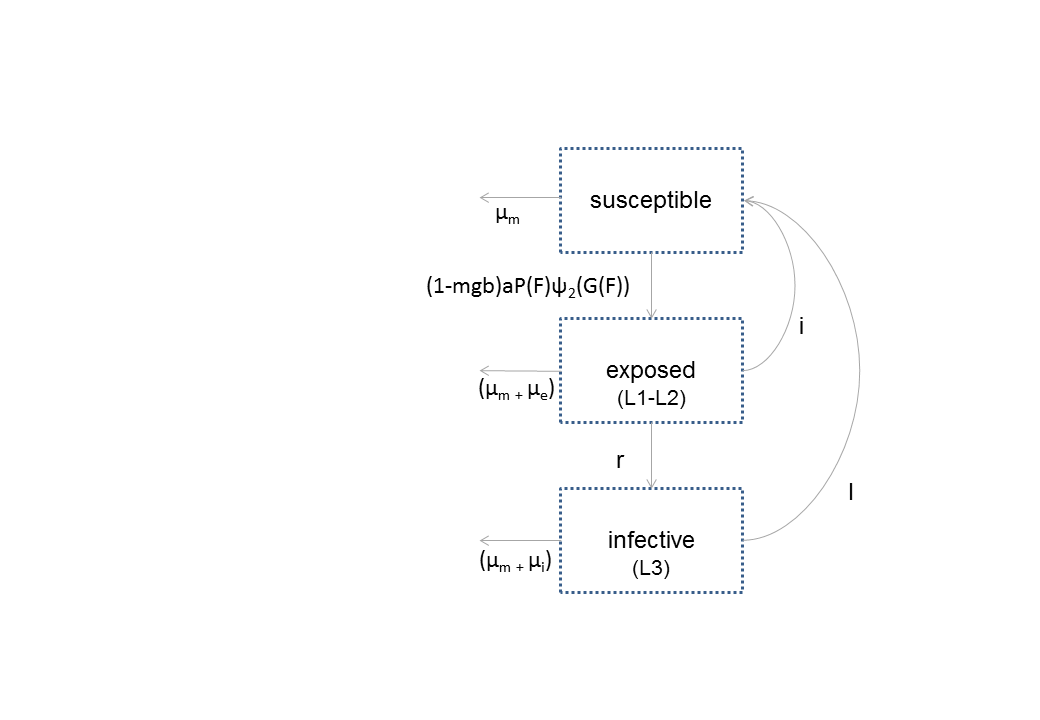
**

**Fig. S1**: Diagram depicting compartments of mosquitoes related to *W. bancrofti* infection status and the plausible transitions between them. Uninfected mosquitoes die at a rate μ_m_ and become infected at a rate depending on the prevalence of infection in humans, the probability of ingesting microfilariae, the biting rate and putatively a midgut barrier, mgb (in the main model, m = 1 - mgb). Exposed mosquitoes may suffer an additional mortality, μ_e_, and could clear infection due to an immune response (i); otherwise ingested microfilariae develop into third stage larvae at rate r. Additional interactions, that are not included in the main model, are that infective mosquitoes may suffer an additional mortality, μ_i_, and could clear infection by loss of L_3_’s during feeding at rate l.

**Figure S2**


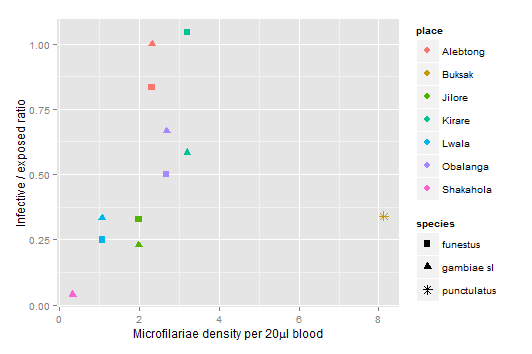


**Fig. S2**: The ratio of field-collected mosquitoes harboring infective (L3) stage larvae to mosquitoes infected but not yet infective (carrying 1^st^ and 2^nd^ stage larvae) against mean microfilariae counts in 20 μl of blood derived from prevalence data [[5](#_ENREF_5),[6](#_ENREF_6),[7](#_ENREF_7),[8](#_ENREF_8)].

**Figure S3**


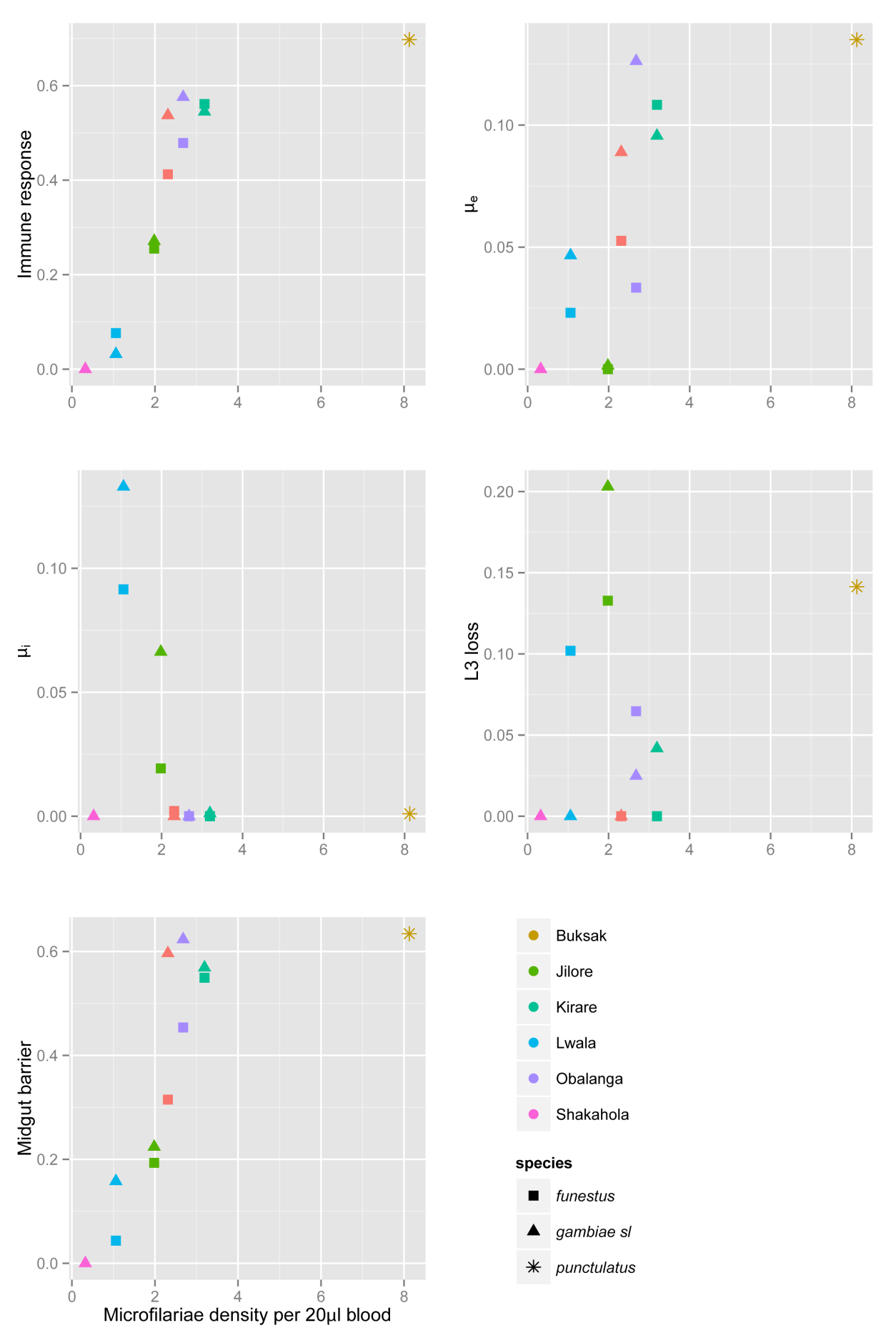


**Fig. S3**: Parameter value estimates for a model with parameters as depicted in Figure S1 fit to ratios of LF susceptible, exposed and infective mosquitoes assuming infection in humans and mosquito population size are stable.

**Sextion III: Modeling long-lasting insecticidal nets (LLINS)**

The effects of LLINs are investigated using the formulae derived by Le Menach et al [[9](#_ENREF_9)] for the feeding cycle duration and probability of survival under varying LLIN coverage levels, and substituting these equations in our expressions for R_0_. We make the simplifying assumption that mosquitoes are fully anthropophilic, but this can be relaxed if species-specific differences in feeding behaviour are of interest. Briefly, mosquitoes can attack a human with probability Q_(0)_; the probability of a human being protected by a bed net depends on the level of coverage, φ. Upon encountering a human protected by a net, a mosquito may feed successfully with probability *s*, be exposed to insecticides and die with probability *d*, or be repelled and start a new feeding cycle with probability *r*. Following a blood meal a mosquito will rest and oviposit before starting another cycle. The duration of the feeding cycle, 1/f_(0)_, is equivalent to the inverse of parameter a, and is τ_1(0)_+ τ_2_, i.e., the duration of the host-seeking phase in the absence of nets and the duration of the resting and oviposition phase. The duration of the feeding cycle in the presence of nets is given by 1/f_(φ)_= τ_1(0)_/(1- φ r)+ τ_2_. The probability of surviving a day in the absence of nets is given by p_(0)_=(p_1(0)_+p_2_)^f(0)^, and p_(φ)_= (p_1(0)_+p_2_W/(1- φ r p_1(0)_)^f(φ)^, where W=1-φ (1-s).

We substitute f_(φ)_ and –ln(p_(φ)_) for the biting rate *a* and mosquito mortality μ_m_. To account for parasite-induced mortality, instead of writing this as an additional mortality we use a single term, so that for malaria-infective mosquitoes μ_mi_=μ_m_+μ_i_= -ln(p_(0)i_), and similarly for LF and co-infected mosquitoes. The equilibrium number of susceptible mosquitoes then becomes Λ /μ_m(φ)_, where μ_m(φ)_ = -ln(p_(φ)_). The combination of larval control and ITNs can then be modelled by using the mortality and biting rates as a function of LLIN coverage and Λ(1-φ)/μ_m(φ)_ for the abundance of mosquitoes. The equilibrium number of susceptible, exposed and infective mosquitoes was solved numerically because an additional mortality term is introduced in the presence of either parasite.

**Section IV: Basic reproductive numbers for malaria and lymphatic filariasis**

*R_0_ for malaria in the absence of lymphatic filariasis*

We use the next-generation matrix (NGM) approach to obtain expressions of the basic reproductive number, R_0_, for malaria and lymphatic filariasis in the absence and presence of the other disease following the methods of Diekmann et al [[10](#_ENREF_10)].

For malaria, using a simplified model with only three compartments for mosquitoes and no terms associated with filariasis, the Jacobian associated with the infection subsystem (*E_h_, I_h_, R_h_, M_es_, M_is_*) is:

$$\Im\mathbf{=}\left(\begin{matrix} \mathbf{-(v}_{\mathbf{h}}\mathbf{+\mu}_{\mathbf{h}}\mathbf{)} & \mathbf{0} & \mathbf{0} & \mathbf{0} & \mathbf{ac} \\ \mathbf{v}_{\mathbf{h}} & \mathbf{-(\gamma}_{\mathbf{h}}\mathbf{+\mu}_{\mathbf{h}}\mathbf{+\delta}_{\mathbf{h}}\mathbf{)} & \mathbf{0} & \mathbf{0} & \mathbf{0} \\ \mathbf{0} & \mathbf{\gamma}_{\mathbf{h}} & \mathbf{-(\mu}_{\mathbf{h}}\mathbf{+\rho}_{\mathbf{h}}\mathbf{)} & \mathbf{0} & \mathbf{0} \\ \mathbf{0} & \frac{\mathbf{abM}_{\mathbf{s}}^{\mathbf{*}}}{\mathbf{N}_{\mathbf{h}}^{\mathbf{*}}} & \frac{\mathbf{a}\tilde{\mathbf{b}}\mathbf{M}_{\mathbf{s}}^{\mathbf{*}}}{\mathbf{N}_{\mathbf{h}}^{\mathbf{*}}} & \mathbf{-(v}_{\mathbf{e}}\mathbf{+\mu}_{\mathbf{m}}\mathbf{)} & \mathbf{0} \\ \mathbf{0} & \mathbf{0} & \mathbf{0} & \mathbf{v}_{\mathbf{e}} & \mathbf{-(\mu}_{\mathbf{m}}\mathbf{+\mu}_{\mathbf{i}}\mathbf{)} \end{matrix}\right)$$

Where M_s_^*^ and N_h_^*^ are as defined in the main text. This Jacobian can be decomposed into two matrices T and Σ, which hold transmission events and transitions between states, respectively.

$$\mathbf{T=}\left(\begin{matrix} \mathbf{0} & \mathbf{0} & \mathbf{0} & \mathbf{0} & \mathbf{ac} \\ \mathbf{0} & \mathbf{0} & \mathbf{0} & \mathbf{0} & \mathbf{0} \\ \mathbf{0} & \mathbf{0} & \mathbf{0} & \mathbf{0} & \mathbf{0} \\ \mathbf{0} & \frac{\mathbf{abM}_{\mathbf{s}}^{\mathbf{*}}}{\mathbf{N}_{\mathbf{h}}^{\mathbf{*}}} & \frac{\mathbf{a}\tilde{\mathbf{b}}\mathbf{M}_{\mathbf{s}}^{\mathbf{*}}}{\mathbf{N}_{\mathbf{h}}^{\mathbf{*}}} & \mathbf{0} & \mathbf{0} \\ \mathbf{0} & \mathbf{0} & \mathbf{0} & \mathbf{0} & \mathbf{0} \end{matrix}\right)$$

$$\mathbf{\Sigma=}\left(\begin{matrix} \mathbf{-(v}_{\mathbf{h}}\mathbf{+\mu}_{\mathbf{h}}\mathbf{)} & \mathbf{0} & \mathbf{0} & \mathbf{0} & \mathbf{0} \\ \mathbf{v}_{\mathbf{h}} & \mathbf{-(\gamma}_{\mathbf{h}}\mathbf{+\mu}_{\mathbf{h}}\mathbf{+\delta}_{\mathbf{h}}\mathbf{)} & \mathbf{0} & \mathbf{0} & \mathbf{0} \\ \mathbf{0} & \mathbf{\gamma}_{\mathbf{h}} & \mathbf{-(\mu}_{\mathbf{h}}\mathbf{+\rho}_{\mathbf{h}}\mathbf{)} & \mathbf{0} & \mathbf{0} \\ \mathbf{0} & \mathbf{0} & \mathbf{0} & \mathbf{-(v}_{\mathbf{e}}\mathbf{+\mu}_{\mathbf{m}}\mathbf{)} & \mathbf{0} \\ \mathbf{0} & \mathbf{0} & \mathbf{0} & \mathbf{v}_{\mathbf{e}} & \mathbf{-(\mu}_{\mathbf{m}}\mathbf{+\mu}_{\mathbf{i}}\mathbf{)} \end{matrix}\right)$$

The NGM with large domain, K_L_, is given by the spectral radius of –T Σ^-1^. This can be reduced by writing a matrix E:

$$\mathbf{E=}\left(\begin{matrix} \mathbf{1} & \mathbf{0} \\ \mathbf{0} & \mathbf{0} \\ \mathbf{0} & \mathbf{0} \\ \mathbf{0} & \mathbf{1} \\ \mathbf{0} & \mathbf{0} \end{matrix}\right)$$

The NGM K is then given by –E’T Σ^-1^E:

$$\mathbf{K=}\left(\begin{matrix} \mathbf{0} & \frac{\mathbf{acv}_{\mathbf{e}}}{\mathbf{(\mu}_{\mathbf{m}}\mathbf{+\mu}_{\mathbf{i}}\mathbf{)}\left(\mathbf{\mu}_{\mathbf{m}}\mathbf{+v}_{\mathbf{e}}\right)} \\ \frac{\mathbf{aM}_{\mathbf{s}}^{\mathbf{*}}\mathbf{v}_{\mathbf{h}}\left(\mathbf{b}\left(\mathbf{\mu}_{\mathbf{h}}\mathbf{+\rho}_{\mathbf{h}}\right)\mathbf{+}\tilde{\mathbf{b}}\mathbf{\gamma}_{\mathbf{h}}\right)}{\mathbf{N}_{\mathbf{h}}^{\mathbf{*}}\left(\mathbf{\mu}_{\mathbf{h}}\mathbf{+\rho}_{\mathbf{h}}\right)\left(\mathbf{\mu}_{\mathbf{h}}\mathbf{+v}_{\mathbf{h}}\right)\left(\mathbf{\gamma}_{\mathbf{h}}\mathbf{+\mu}_{\mathbf{h}}\mathbf{+\delta}_{\mathbf{h}}\right)} & \mathbf{0} \end{matrix}\right)$$

and R_0,m_ = ρ(K)^2^, the spectral radius or dominant eigenvalue of the next-generation matrix, squared to reflect the transmission from human to human:

|  | $R_{0,m}=\frac{a^{2}c M_{s}^{*}\text{ v}_{e}\text{ v}_{h} \left(b\left(\mu_{h}+\rho_{h}\right)+\tilde{b}\gamma_{h}\right)}{N_{h}^{*}\left(\mu_{h}+\rho_{h}\right)(\mu_{m}+\mu_{i})\left(\mu_{m}+ v_{e}\right)\left(\mu_{h}+v_{h}\right)\left(\gamma_{h}+\mu_{h}+\delta_{h}\right)}$ | (S4.1) |
| --- | --- | --- |

*R_0_ for lymphatic filariasis in the absence of malaria*

Following the same logic for lymphatic filariasis with infection subsystem, (*W,F, M_se_, M_si_*), we write:

$$\begin{matrix} \mathbf{T=}\left(\begin{matrix} \mathbf{0} & \mathbf{0} & \mathbf{0} & \mathbf{ }\frac{\mathbf{\psi}_{\mathbf{1}}\mathbf{aL}_{\mathbf{3}}}{\mathbf{N}_{\mathbf{h}}^{\mathbf{*}}} \\ \mathbf{0} & \mathbf{0} & \mathbf{0} & \mathbf{0} \\ \mathbf{0} & \frac{\mathbf{e}^{\mathbf{-k}_{\mathbf{\rho}}\mathbf{k}_{\mathbf{\psi}}}\left(\mathbf{e}^{\mathbf{k}_{\mathbf{\rho}}\mathbf{k}_{\mathbf{\psi}}}\mathbf{-1}\right)}{\mathbf{k}_{\mathbf{\rho}}}\mathbf{m aM}_{\mathbf{s}}^{\mathbf{*}} & \mathbf{0} & \mathbf{0} \\ \mathbf{0} & \mathbf{0} & \mathbf{0} & \mathbf{0} \end{matrix}\right) \\ \\ \mathbf{\Sigma=}\left(\begin{matrix} \mathbf{-\mu}_{\mathbf{w}} & \mathbf{0} & \mathbf{0} & \mathbf{0} \\ \mathbf{\alpha} & \mathbf{-\mu}_{\mathbf{f}} & \mathbf{0} & \mathbf{0} \\ \mathbf{0} & \mathbf{0} & \mathbf{-(\mu}_{\mathbf{m}}\mathbf{+\mu}_{\mathbf{e}}\mathbf{+r+i)} & \mathbf{0} \\ \mathbf{0} & \mathbf{0} & \mathbf{r} & \mathbf{-\mu}_{\mathbf{m}} \end{matrix}\right) \end{matrix}$$

The NGM K is –E’T Σ^-1^E and R_0,f_ = ρ(K)^k^. For R_0_ to be consistent across models of the same parasite that differ in the number of life stages explicitly modelled, the spectral radius is raised to the power *k*, where equals the number of life stages. For helminth models this is often three [[11](#_ENREF_11),[12](#_ENREF_12)], but because we do not explicitly model the larval stage in mosquitoes here *k* is two, and R_0,f_ is:

|  | $R_{0,f}=\frac{a^{2}\text{e}^{-\text{k}_{\text{ρ}}\text{k}_{\text{ψ}}}\left(\text{e}^{\text{k}_{\text{ρ}}\text{k}_{\text{ψ}}}-1\right)mr\alpha L_{3}M_{s}^{*}\psi_{1}}{\text{k}_{\text{ρ}}N_{h}^{*}\mu_{f}\mu_{m}\left(\text{i}+r+\mu_{e}+\mu_{m}\right)\mu_{w}}$ | (S4.2) |
| --- | --- | --- |

*R_0_ for malaria in the presence of lymphatic filariasis:*

We use the same approach for the full model with infection subsystem (*E_h_, I_h_, R_h_, M_es_, M_ee_, M_ei_, M_is_, M_ie_, M_ii_*):

$$\mathbf{T=}\left(\begin{matrix} \mathbf{0} & \mathbf{0} & \mathbf{0} & \mathbf{0} & \mathbf{0} & \mathbf{0} & \mathbf{ac} & \mathbf{ac} & \mathbf{ac} \\ \mathbf{0} & \mathbf{0} & \mathbf{0} & \mathbf{0} & \mathbf{0} & \mathbf{0} & \mathbf{0} & \mathbf{0} & \mathbf{0} \\ \mathbf{0} & \mathbf{0} & \mathbf{0} & \mathbf{0} & \mathbf{0} & \mathbf{0} & \mathbf{0} & \mathbf{0} & \mathbf{0} \\ \mathbf{0} & \frac{\mathbf{abM}_{\mathbf{ss}}^{\mathbf{*}}}{\mathbf{N}_{\mathbf{h}}^{\mathbf{*}}} & \frac{\mathbf{a}\tilde{\mathbf{b}}\mathbf{M}_{\mathbf{ss}}^{\mathbf{*}}}{\mathbf{N}_{\mathbf{h}}^{\mathbf{*}}} & \mathbf{0} & \mathbf{0} & \mathbf{0} & \mathbf{0} & \mathbf{0} & \mathbf{0} \\ \mathbf{0} & \frac{\mathbf{abM}_{\mathbf{se}}^{\mathbf{*}}}{\mathbf{N}_{\mathbf{h}}^{\mathbf{*}}} & \frac{\mathbf{a}\tilde{\mathbf{b}}\mathbf{M}_{\mathbf{se}}^{\mathbf{*}}}{\mathbf{N}_{\mathbf{h}}^{\mathbf{*}}} & \mathbf{0} & \mathbf{0} & \mathbf{0} & \mathbf{0} & \mathbf{0} & \mathbf{0} \\ \mathbf{0} & \frac{\mathbf{abM}_{\mathbf{si}}^{\mathbf{*}}}{\mathbf{N}_{\mathbf{h}}^{\mathbf{*}}} & \frac{\mathbf{a}\tilde{\mathbf{b}}\mathbf{M}_{\mathbf{si}}^{\mathbf{*}}}{\mathbf{N}_{\mathbf{h}}^{\mathbf{*}}} & \mathbf{0} & \mathbf{0} & \mathbf{0} & \mathbf{0} & \mathbf{0} & \mathbf{0} \\ \mathbf{0} & \mathbf{0} & \mathbf{0} & \mathbf{0} & \mathbf{0} & \mathbf{0} & \mathbf{0} & \mathbf{0} & \mathbf{0} \\ \mathbf{0} & \mathbf{0} & \mathbf{0} & \mathbf{0} & \mathbf{0} & \mathbf{0} & \mathbf{0} & \mathbf{0} & \mathbf{0} \\ \mathbf{0} & \mathbf{0} & \mathbf{0} & \mathbf{0} & \mathbf{0} & \mathbf{0} & \mathbf{0} & \mathbf{0} & \mathbf{0} \end{matrix}\right)$$

$$\mathbf{E=}\left(\begin{matrix} \mathbf{1} & \mathbf{0} & \mathbf{0} & \mathbf{0} \\ \mathbf{0} & \mathbf{0} & \mathbf{0} & \mathbf{0} \\ \mathbf{0} & \mathbf{0} & \mathbf{0} & \mathbf{0} \\ \mathbf{0} & \mathbf{1} & \mathbf{0} & \mathbf{0} \\ \mathbf{0} & \mathbf{0} & \mathbf{1} & \mathbf{0} \\ \mathbf{0} & \mathbf{0} & \mathbf{0} & \mathbf{1} \\ \mathbf{0} & \mathbf{0} & \mathbf{0} & \mathbf{0} \\ \mathbf{0} & \mathbf{0} & \mathbf{0} & \mathbf{0} \\ \mathbf{0} & \mathbf{0} & \mathbf{0} & \mathbf{0} \end{matrix}\right)$$

K is then given by –E’T Σ^-1^E and R_0_ = ρ(K)^2^, the spectral radius or dominant eigenvalue of the next generation matrix (squared to reflect the increase from human-human) which becomes an unwieldy expression and is not reproduced here. In the absence of μ_e_, i.e., mortality due to *W. bancrofti* infection, the expression simplifies to:

|  | $R_{0,m}=\frac{a^{2}c (M_{ss}^{*}+M_{se}^{*}+M_{si}^{*})\text{ v}_{e}\text{ v}_{h} \left(b\left(\mu_{h}+\rho_{h}\right)+\tilde{b}\gamma_{h}\right)}{N_{h}^{*}\left(\mu_{h}+\rho_{h}\right)(\mu_{m}+\mu_{i})\left(\mu_{m}+ v_{e}\right)\left(\mu_{h}+v_{h}\right)\left(\gamma_{h}+\mu_{h}+\delta_{h}\right)}$ | (S4.3) |
| --- | --- | --- |

Although we do not have explicit expressions for the equilibrum values of the proportions of mosquitoes that are susceptible, exposed and infectious with LF ($M_{\mathrm{ss}}^{*}$, $M_{\mathrm{se}}^{*}$, and $M_{\mathrm{si}}^{*}$), we can evaluate them numerically to calculate *R_0,m_* for particular sets of parameter values.

*R_0_ for lymphatic filariasis in the presence of malaria:*

We use the same approach for the full model with infection subsystem (*W,F, M_se_, M_ee_, M_ie_, M_si_, M_ei_, M_ii_*):

$$\mathbf{T=}\left(\begin{matrix} \mathbf{0} & \mathbf{0} & \mathbf{0} & \mathbf{0} & \mathbf{0} & \frac{\mathbf{\psi}_{\mathbf{1}}\mathbf{aL}_{\mathbf{3}}}{\mathbf{N}_{\mathbf{h}}^{\mathbf{*}}} & \frac{\mathbf{\psi}_{\mathbf{1}}\mathbf{aL}_{\mathbf{3}}}{\mathbf{N}_{\mathbf{h}}^{\mathbf{*}}} & \frac{\mathbf{\psi}_{\mathbf{1}}\mathbf{aL}_{\mathbf{3}}}{\mathbf{N}_{\mathbf{h}}^{\mathbf{*}}} \\ \mathbf{0} & \mathbf{0} & \mathbf{0} & \mathbf{0} & \mathbf{0} & \mathbf{0} & \mathbf{0} & \mathbf{0} \\ \mathbf{0} & \frac{\mathbf{e}^{\mathbf{-k}_{\mathbf{\rho}}\mathbf{k}_{\mathbf{\psi}}}\left(\mathbf{e}^{\mathbf{k}_{\mathbf{\rho}}\mathbf{k}_{\mathbf{\psi}}\mathbf{m}}\mathbf{-1}\right)}{\mathbf{k}_{\mathbf{\rho}}}\mathbf{maM}_{\mathbf{ss}}^{\mathbf{*}} & \mathbf{0} & \mathbf{0} & \mathbf{0} & \mathbf{0} & \mathbf{0} & \mathbf{0} \\ \mathbf{0} & \frac{\mathbf{e}^{\mathbf{-k}_{\mathbf{\rho}}\mathbf{k}_{\mathbf{\psi}}}\left(\mathbf{e}^{\mathbf{k}_{\mathbf{\rho}}\mathbf{k}_{\mathbf{\psi}}\mathbf{m}}\mathbf{-1}\right)}{\mathbf{k}_{\mathbf{\rho}}}\mathbf{maM}_{\mathbf{es}}^{\mathbf{*}} & \mathbf{0} & \mathbf{0} & \mathbf{0} & \mathbf{0} & \mathbf{0} & \mathbf{0} \\ \mathbf{0} & \frac{\mathbf{e}^{\mathbf{-k}_{\mathbf{\rho}}\mathbf{k}_{\mathbf{\psi}}}\left(\mathbf{e}^{\mathbf{k}_{\mathbf{\rho}}\mathbf{k}_{\mathbf{\psi}}\mathbf{m}}\mathbf{-1}\right)}{\mathbf{k}_{\mathbf{\rho}}}\mathbf{maM}_{\mathbf{is}}^{\mathbf{*}} & \mathbf{0} & \mathbf{0} & \mathbf{0} & \mathbf{0} & \mathbf{0} & \mathbf{0} \\ \mathbf{0} & \mathbf{0} & \mathbf{0} & \mathbf{0} & \mathbf{0} & \mathbf{0} & \mathbf{0} & \mathbf{0} \\ \mathbf{0} & \mathbf{0} & \mathbf{0} & \mathbf{0} & \mathbf{0} & \mathbf{0} & \mathbf{0} & \mathbf{0} \\ \mathbf{0} & \mathbf{0} & \mathbf{0} & \mathbf{0} & \mathbf{0} & \mathbf{0} & \mathbf{0} & \mathbf{0} \end{matrix}\right)$$

$$\sum\mathbf{=}\left(\begin{matrix} \mathbf{-\mu}_{\mathbf{w}} & \mathbf{0} & \mathbf{0} & \mathbf{0} & \mathbf{0} & \mathbf{0} & \mathbf{0} & \mathbf{0} \\ \mathbf{\alpha} & \mathbf{-\mu}_{\mathbf{f}} & \mathbf{0} & \mathbf{0} & \mathbf{0} & \mathbf{0} & \mathbf{0} & \mathbf{0} \\ \mathbf{0} & \mathbf{0} & \mathbf{-a}\left(\mathbf{b}\frac{\mathbf{I}_{\mathbf{h}}}{\mathbf{N}_{\mathbf{h}}^{\mathbf{*}}}\mathbf{+}\tilde{\mathbf{b}}\frac{\mathbf{R}_{\mathbf{h}}}{\mathbf{N}_{\mathbf{h}}^{\mathbf{*}}}\right)\mathbf{-r-i-\mu}_{\mathbf{m}}\mathbf{-\mu}_{\mathbf{e}} & \mathbf{0} & \mathbf{0} & \mathbf{0} & \mathbf{0} & \mathbf{0} \\ \mathbf{0} & \mathbf{0} & \mathbf{a}\left(\mathbf{b}\frac{\mathbf{I}_{\mathbf{h}}}{\mathbf{N}_{\mathbf{h}}^{\mathbf{*}}}\mathbf{+}\tilde{\mathbf{b}}\frac{\mathbf{R}_{\mathbf{h}}}{\mathbf{N}_{\mathbf{h}}^{\mathbf{*}}}\right) & \mathbf{-r-i-v}_{\mathbf{e}}\mathbf{-\mu}_{\mathbf{m}}\mathbf{-\mu}_{\mathbf{e}} & \mathbf{0} & \mathbf{0} & \mathbf{0} & \mathbf{0} \\ \mathbf{0} & \mathbf{0} & \mathbf{0} & \mathbf{v}_{\mathbf{e}} & \mathbf{-r-i-\mu}_{\mathbf{m}}\mathbf{-\mu}_{\mathbf{e}}\mathbf{-\mu}_{\mathbf{i}} & \mathbf{0} & \mathbf{0} & \mathbf{0} \\ \mathbf{0} & \mathbf{0} & \mathbf{r} & \mathbf{0} & \mathbf{0} & \mathbf{-a}\left(\mathbf{b}\frac{\mathbf{I}_{\mathbf{h}}}{\mathbf{N}_{\mathbf{h}}^{\mathbf{*}}}\mathbf{+}\tilde{\mathbf{b}}\frac{\mathbf{R}_{\mathbf{h}}}{\mathbf{N}_{\mathbf{h}}^{\mathbf{*}}}\right)\mathbf{-\mu}_{\mathbf{m}} & \mathbf{0} & \mathbf{0} \\ \mathbf{0} & \mathbf{0} & \mathbf{0} & \mathbf{r} & \mathbf{0} & \mathbf{a}\left(\mathbf{b}\frac{\mathbf{I}_{\mathbf{h}}}{\mathbf{N}_{\mathbf{h}}^{\mathbf{*}}}\mathbf{+}\tilde{\mathbf{b}}\frac{\mathbf{R}_{\mathbf{h}}}{\mathbf{N}_{\mathbf{h}}^{\mathbf{*}}}\right) & \mathbf{-\mu}_{\mathbf{m}}\mathbf{-v}_{\mathbf{e}} & \mathbf{0} \\ \mathbf{0} & \mathbf{0} & \mathbf{0} & \mathbf{0} & \mathbf{r} & \mathbf{0} & \mathbf{v}_{\mathbf{e}} & \mathbf{-\mu}_{\mathbf{m}}\mathbf{-\mu}_{\mathbf{i}} \end{matrix}\right)$$

In the absence of *Plasmodium*-induced vector mortality R_0_ then becomes:

|  |  | (S4.4) |
| --- | --- | --- |

which is similar to the expression of R_0_ in the absence of malaria, S4.2. With mortality included, the expression likewise becomes unwieldy. Similar to the basic reproductive number for malaria, although we do not have explicit expressions for the equilibrum values of the proportions of mosquitoes that are susceptible, exposed and infectious with malaria ($M_{\mathrm{ss}}^{*}$, $M_{\mathrm{es}}^{*}$, and $M_{\mathrm{is}}^{*}$), we can evaluate them numerically to calculate *R_0,f_* for particular sets of parameter values.

**References**

1. Anderson R, May R (1985) Helminth infections of humans: mathematical models, population dynamics, and control. Advances in Parasitology 24: 1-101.

2. Chitnis N, Cushing J, Hyman J (2006) Bifurcation analysis of a mathematical model for malaria transmission. SIAM Journal on Applied Mathematics 67: 24-45.

3. Lindsay S, Denham D (1986) The ability of *Aedes aegypti* mosquitoes to survive and transmit infective larvae of *Brugia pahangi* over successive blood meals. Journal of Helminthology 60: 159-168.

4. Bryan J, Southgate B (1988) Factors affecting transmission of Wuchereria bancrofti by anopheline mosquitoes. 2. Damage to ingested microfilariae by mosquito foregut armatures and development of filarial larvae in mosquitoes. Transactions of the Royal Society of Tropical Medicine and Hygiene 82: 138-145.

5. Simonsen P, Pedersen E, Rwegoshora R, Malecela M, Derua Y, et al. (2010) Lymphatic filariasis control in Tanzania: effect of repeated mass drug administration with ivermectin and albendazole on infection and transmission. PloS Neglected Tropical Diseases 4: e696.

6. Burkot T, Molineaux L, Graves P, Paru R, Battistutta D, et al. (1990) The prevalence of naturally acquired multiple infections of *Wuchereria bancrofti* and human malarias in anophelines. Parasitology 100: 369-375.

7. Onapa A, Simonsen P, Pedersen E, Okello D (2001) Lymphatic filariasis in Uganda: baseline investigations in Lira, Soroti and Katakwi districts. Transactions of the Royal Society of Tropical Medicine and Hygiene 95: 161-167.

8. Muturi E, Mbogo C, Mwangangi J, Ng'ang'a Z, Kabiru E, et al. (2006) Concomitant infections of *Plasmodium falciparum* and *Wuchereria bancrofti* on the Kenyan coast. Filaria Journal 5: 8.

9. Le Menach A, Takala S, McKenzie F, Perisse A, Harris A, et al. (2007) An elaborated feeding cycle model for reductions in vectorial capacity of night-biting mosquitoes by insecticide-treated nets. Malaria Journal 6: 10.

10. Diekmann O, Heesterbeek J, Roberts M (2010) The construction of next-generation matrices for compartmental epidemic models. Journal of the Royal Society Interface 7: 873-885.

11. Heesterbeek J, Roberts M (1995) Threshold quantities for helminth infections. Journal of Mathematical Biology 33: 415-434.

12. Slater H, Gambhir M, Parham P, Michael E (2013) Modelling co-infection with malaria and lymphatic filariasis. PloS Computational Biology 9: e1003096.
